# High-resolution yeast actin structures indicate the molecular mechanism of actin filament stiffening by cations

**DOI:** 10.1038/s42004-024-01243-x

**Published:** 2024-07-30

**Authors:** Xiao-Ping Xu, Wenxiang Cao, Mark F. Swift, Nandan G. Pandit, Andrew E. Huehn, Charles V. Sindelar, Enrique M. De La Cruz, Dorit Hanein, Niels Volkmann

**Affiliations:** 1 Scintillon Institute, 6868 Nancy Ridge Drive, San Diego, CA 92121 USA; 2 Department of Molecular Biophysics and Biochemistry, Yale University, New Haven, CT 06520 USA; 3 Department of Chemistry and Biochemistry and Department of Biological Engineering, University of California, Santa Barbara, CA 93106 USA; 4 Department of Biological Engineering, Department of Electrical and Computer Engineering, Biomolecular Science and Engineering Program, University of California, Santa Barbara, CA 93106 USA

**Keywords:** Cryoelectron microscopy, Molecular modelling, Cytoskeletal proteins

## Abstract

Actin filament assembly and the regulation of its mechanical properties are fundamental processes essential for eukaryotic cell function. Residue E167 in vertebrate actins forms an inter-subunit salt bridge with residue K61 of the adjacent subunit. *Saccharomyces cerevisiae* actin filaments are more flexible than vertebrate filaments and have an alanine at this position (A167). Substitution of this alanine for a glutamic acid (A167E) confers *Saccharomyces cerevisiae* actin filaments with salt-dependent stiffness similar to vertebrate actins. We developed an optimized cryogenic electron microscopy workflow refining sample preparation and vitrification to obtain near-atomic resolution structures of wild-type and A167E mutant *Saccharomyces cerevisiae* actin filaments. The difference between these structures allowed us to pinpoint the potential binding site of a filament-associated cation that controls the stiffness of the filaments in vertebrate and A167E *Saccharomyces cerevisiae* actins. Through an analysis of previously published high-resolution reconstructions of vertebrate actin filaments, along with a newly determined high-resolution vertebrate actin structure in the absence of potassium, we identified a unique peak near residue 167 consistent with the binding of a magnesium ion. Our findings show how magnesium can contribute to filament stiffening by directly bridging actin subunits and allosterically affecting the orientation of the DNase-I binding loop of actin, which plays a regulatory role in modulating actin filament stiffness and interactions with regulatory proteins.

## Abstract (web summary)

Actin filament assembly and the regulation of its mechanical properties are fundamental processes essential for eukaryotic cell function, however, the molecular mechanisms that govern the mechanical properties of the actin filaments formed from different species are not fully understood. Here, the authors report high-resolution cryo-EM reconstructions of yeast actin from *Saccharomyces cerevisiae* and propose how the mechanism of the stiffening of the actin filament is affected by magnesium cations.

## Introduction

Actin filaments play central roles in a variety of cellular functions in eukaryotes, including cell motility, cell division, determination of cellular shape, and provision of mechanical strength and integrity^1,2^. The force-generating and mechanosensing properties of actin are governed by the filament’s mechanical properties, such as the bending^3,4^ and torsional stiffness^5^. Actin filaments are polyampholytes that contain both positively and negatively charged regions, and the mechanical properties of vertebrate actin filaments, in particular, depend on solution conditions, specifically monovalent and divalent cations^6,7^.

Actin filaments from *Saccharomyces cerevisiae* (from here on referred to as yeast) are distinct from all other characterized actins in that they do not display salt-dependent stiffness, and are overall much more flexible than their vertebrate actin filament counterparts at physiological salt concentrations^6,7^. Vertebrate actin carries a glutamic acid at residue 167 (E167), and in most actin filament structures with better than 5-Å resolution, at least one of the side-chain oxygen atoms of residue E167 is within 5 Å of the side-chain nitrogen of residue K61 in the adjacent actin subunit of the filament, likely forming a stabilizing ionic interaction^8^ or salt bridge. Because residue 167 in yeast actin is an alanine and, therefore, not able to form that interaction, it is tempting to assume that the E167–K61 salt bridge is responsible for the higher stiffness of vertebrate filaments. Consistent with this prediction, if the alanine at residue 167 in yeast is replaced by a glutamic acid to form the A167E mutant, the mutant yeast actin displays similar salt-dependent stiffness behavior as vertebrate actin^6^. However, this does not explain the salt dependence of the stiffness or why the A167E mutant actin is less stiff at low salt concentrations than the wild-type filaments^6^. How solution salts, particularly the physiologically relevant cation, Mg^2+^, modulate actin filament stiffness has remained elusive^7^, and high-resolution structural details are required to elucidate the underlying origins and molecular mechanisms.

Because of the relative ease of mutating actin residues in yeast, it is possible to investigate the effect of single residue substitutions on the properties of actin, including E167. Despite the ability to introduce mutations, yeast actin has not been widely used for actin filament structure determination. The reason is that it has been difficult to generate samples amenable to high-resolution reconstructions. Low-resolution wild-type yeast actin filament structures were derived by electron microscopy of negatively stained filaments^9,10^, and cryogenic electron microscopy (cryo-EM) reconstructions of the A167E mutant were restricted to about 10-Å resolution^11^. We developed an optimized workflow refining sample preparation, vitrification, and image processing strategies. This optimized workflow enabled us to achieve near-atomic resolution structures of filamentous wild-type yeast actin and two conformations of filamentous yeast actin carrying the A167E mutation. Additionally, we determined a 2.27-Å resolution structure of vertebrate muscle actin in the absence of potassium and developed improved sharpening protocols that we applied to enhance previously published high-resolution reconstructions of vertebrate actin spanning multiple nucleotide and salt conditions^12–15^. We combine the insights gained from the yeast actin structures and the vertebrate actin reconstructions to propose how magnesium cations contribute to actin filament stiffening.

## Results

### Structures of wild-type and A167E mutant yeast actin filaments

**Fig. 1:**
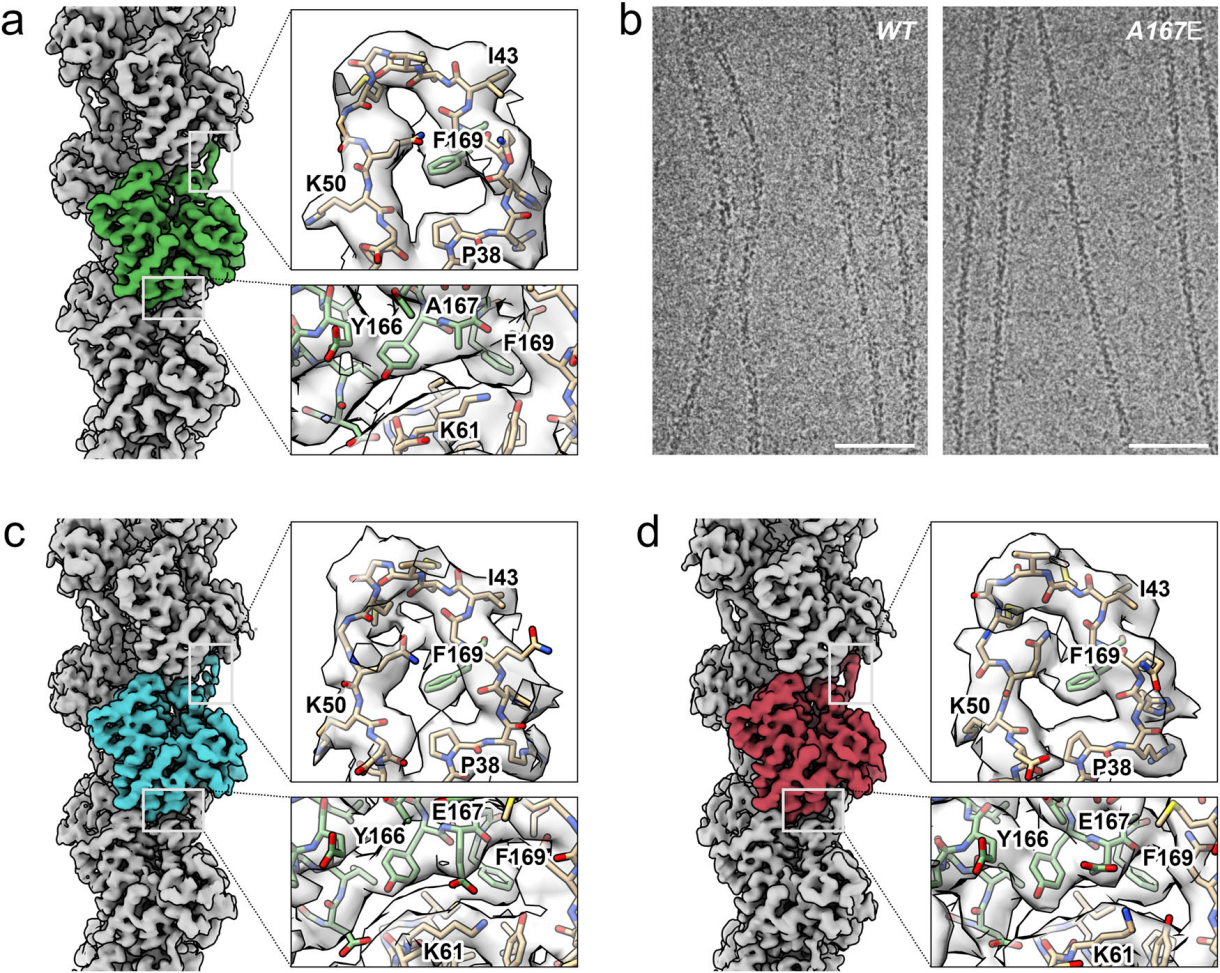
High-resolution structures of wild-type and mutant yeast actin. **a** Reconstruction of wild-type yeast actin with a single actin subunit highlighted in green. **b** Representative micrographs of wild-type (WT, left) and A167E mutant (A167E, right) yeast actin filaments. The scale bars represent 50 nm. **c**, **d** Two reconstructions of A167E mutant yeast actin, one resembling wild-type yeast actin (A167E^WT^) (**c**) and another resembling rabbit vertebrate actin (A167E^RA^) (**d**). In each case, a single actin subunit is highlighted in light blue (A167E^WT^) and in red (A167E^RA^). For **a**, **c**, and **d**, the upper inset shows the fit in the D-loop region, and the lower inset shows the fit at the long-pitch subunit interface near residue E167.

An optimized cryo-EM workflow involving refined sample preparation and vitrification enabled the acquisition of yeast actin filament data sets preserved at near-atomic resolution. We obtained structures of filamentous wild-type yeast actin and two conformations of filamentous yeast actin carrying the A167E mutation (3.83–4.43 Å resolution, Fig. 1a, c, d, Supplementary Fig. S1, Table 1). The two types of yeast filaments exhibit different stiffness behaviors^6^. On the one hand, wild-type yeast filaments are flexible, and their bending stiffness is not affected by solution salts^6^. On the other hand, the A167E yeast mutation, which replaces an alanine in the yeast sequence with a glutamic acid found in vertebrate actins, displays a similar salt-dependent stiffening as vertebrate actin filaments^6^. Our micrographs confirm this observation, as many filaments in the mutant data appear much straighter than those of the wild-type data (Fig. 1b). The estimates for the helical parameters of all three yeast density maps match with a rise of 27.5 ± 0.03 Å per subunit and a twist of −167.2 ± 0.02° (Table 1). These values are consistent with previous measurements^11^, and while there is no statistical difference in rise if compared to vertebrate actin filaments (rise 27.5 Å, twist −166.6°), there is a statistically highly significant 0.6° difference in twist (*p-*value < 10^−15^). The cumulative impact of this 0.6° twist difference leads to an almost 5% increase in the cross-over distance within the yeast actin helix compared to vertebrate actins. This change can have substantial effects on actin function in the cell, influencing factors like myosin-based motility, the density of crosslinkers in actin bundles, and interaction with severing regulatory proteins, all of which have been linked to the cross-over distance^16–18^.

**Table 1:** Data collection, model statistics, and validation

Reconstruction	*Yeast WT*	*A167E*^*WT*^	*A167E*^*RA*^	*Rabbit (no K*^+^*)*
***Data collection***				
Microscope	Titan Krios	Titan Krios	Titan Krios	Titan Krios
Voltage [kV]	300	300	300	300
Detector	Falcon II	Falcon II	Falcon II	Gatan K3
Magnification	75,000	75,000	75,000	105,000
Total exposure [e^−^/Å^2^]	60	60	60	36.4
Pixel size [Å]	1.035	1.035	1.035	0.829
Defocus range [μm]	−0.8 to −2.8	−0.8 to −2.8	−0.8 to −2.8	−1.2 to −2.8
***Data processing***				
Images used	1051	1286	1286	8243
Initial segments	416,447	742,393	742,393	6,024,972
Final segments	238,930	170,753	195,534	5,920,217
*Helical symmetry*				
Rise [Å]	27.43	27.48	27.49	27.51
Twist [°]	−167.23	−167.22	−167.25	−166.47
Resolution [Å]	4.43	3.98	3.83	2.27
FSC threshold	0.143	0.143	0.143	0.143
Sharpening B-factor [Å^2^]	−154.1	−136.8	−116.5	−68.4
***Refinement***				
Initial models [PDB IDs]	5onv	5onv	5onv	5onv
Model resolution [Å]	4.53	4.05	3.91	2.34
FSC threshold	0.5	0.5	0.5	0.5
*RMSD deviations*				
Bond lengths [Å]	0.008	0.008	0.005	0.005
Bond angles [°]	0.761	0.916	0.748	0.626
Mean B-factor [Å^2^]	150.4	65.5	78.5	47.3
***Validation***				
MolProbity score	1.61	1.51	1.29	1.19
Clash score	5.94	6.77	4.87	4.07
Rotamer outliers [%]	0.00	0.00	0.00	0.00
*Ramachandran plot*				
Favored [%]	96.0	97.3	97.8	99.2
Allowed [%]	4.0	2.7	2.2	0.8
Disallowed [%]	0.0	0.0	0.0	0.0
CABLAM outliers [%]	0.27	0.55	1.37	0.00
Masked correlation	0.78	0.80	0.83	0.88
EMRinger score	2.04	2.29	2.41	5.36
*Q* score	0.48	0.50	0.52	0.79
***Deposition codes***				
Density map (EMDB)	EMD-41279	EMD-41273	EMD-41274	EMD-43763
Model (PDB)	8TI3	8THX	8THY	8W36

### Wild-type yeast actin filaments display subtle structural differences from vertebrate actin

**Fig. 2:**
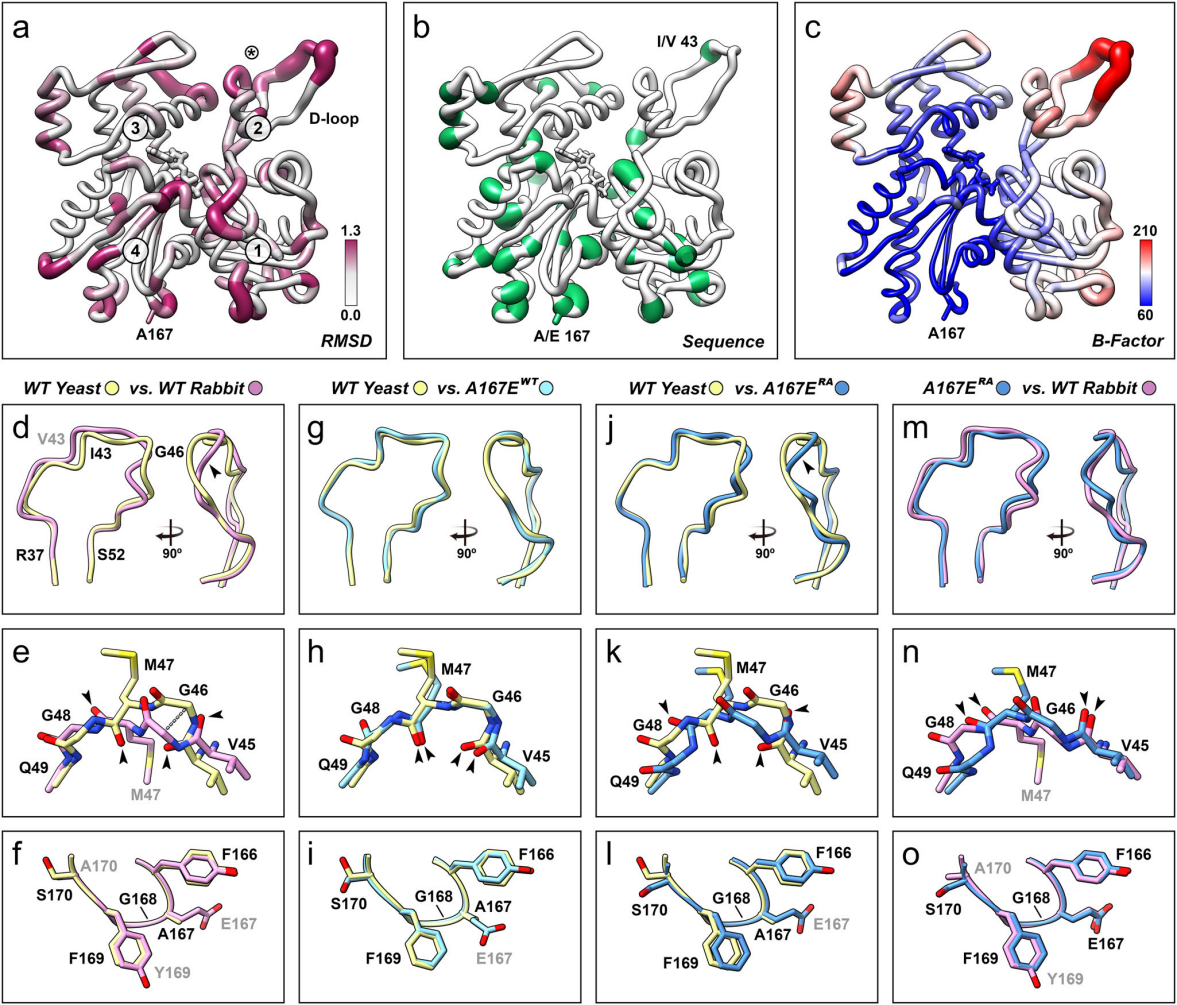
Comparison of actin filament structures. **a** Root-mean-square deviation (RMSD) in Ångstrom between wild-type yeast and rabbit skeletal actin (PDB code 5ONV) mapped onto the structure of wild-type yeast actin. The actin subdomains 1–4 are indicated. Residue A167 and the D-loop are labeled. The asterisk marks the position of A167 of the long-pitch neighbor. **b** Sequence differences between yeast and rabbit actin mapped onto the structure of wild-type yeast actin. Divergent residues are marked in green. Residue 43 and residue 167 are labeled. **c** Temperature factor of wild-type yeast actin mapped onto the wild-type yeast actin structure. Units are in Å^2^. Residue A167 is labeled. **d**–**f** Comparison of wild-type yeast and rabbit actin filament models in the D-loop (**d**, **e**) and 167 regions (**f**). **g–i** Comparison of wild-type yeast and A167E^WT^ actin filament models in the D-loop (**g**, **h**) and 167 region (**i**). The dashed line in **e** indicates the largest α-carbon distance in the D-loop at residue G46. **j**–**l** Comparison of wild-type yeast and A167E^RA^ actin filament models in the D-loop (**j**, **k**) and 167 regions (**l**). **m**–**o** Comparison of rabbit and A167E^WT^ actin filament models in the D-loop (**m**, **n**) and 167 regions (**o**). For panels **d**–**o**, rabbit wild-type actin is shown in pink; yeast wild-type actin is shown in yellow; yeast A167E^WT^ is shown in light blue; yeast A167^RA^ is shown in blue. For the D-loop comparisons **d**, **g**, **j**, **m**, two orthogonal views of smooth representations of the Cα traces are shown. Yeast residues are labeled in black, rabbit residues in gray, and Cα distances over 2.2 Å are marked by arrowheads. For the D-loop details **e**, **h**, **k**, **n** the directions of diverging peptide bonds are marked by arrowheads. For the details of the 167 regions **f**, **I**, **l**, **o**, diverging rabbit wild-type residues and residue E167 of the A167E mutant are labeled in gray, the remaining residues are labeled in black.

The level of sequence similarity between vertebrate rabbit skeletal muscle actin and wild-type yeast actin is 87%. As anticipated, the subunit structures are almost indistinguishable due to this high degree of similarity. When the monomer structures are globally aligned, the local α-carbon root-mean-square deviations (rmsd) do not exceed 2.3 Å anywhere in the structure and are significantly below 1 Å for most of it (Fig. 2a). Regions that contain sequence deviations (Fig. 2b) generally correlate with regions of elevated rmsd values, including the region near residue 167. The highest deviations are present in the DNase-I binding loop (D-loop, residues 38–52) located in subdomain 2 of actin, even though there is only a single conservative sequence deviation in this region (I43 in yeast versus V43 in vertebrate actin). The temperature factors of the refined yeast actin model in this region are also significantly higher compared to the rest of the structure (Fig. 2c).

The D-loop is one of the most flexible elements within actin filaments. Findings from mutational crosslinking experiments, coupled with electron paramagnetic resonance^19^ and molecular dynamics data^20^, indicate a pronounced level of dynamic plasticity in this region. This plasticity manifests itself as detectable polymorphism in low-resolution cryo-EM studies^11,21^, although at higher resolution, the D-loop either appears partially disordered^12^ or as a mix of limited conformational states^22,23^. The D-loop has been linked to the regulation of bending stiffness by coarse-grained molecular dynamics simulations that have implicated D-loop dynamics in filament bending^24^, and cross-linking experiments have shown that D-loop flexibility is essential for filament stability^25^.

The overall orientation of the D-loop in yeast actin filaments is similar to that of the rabbit muscle actin D-loop in the Mg^2+^ ADP state^23,26–28^, but does exhibit a slight reorientation between residues 43 and 49 (Fig. 2d) caused by peptide flips at residues G46 and G48 (Fig. 2e). The flips lead to an α-carbon shift of 2.2 Å at residue G46, the largest α-carbon shift in the entire monomer. Due to insufficiently defined density in this region, not all D-loop side chains can be accurately positioned. Nevertheless, it appears that the side chains do generally point in the same directions as in vertebrate actin filaments, except for residue M47, which points in the opposite direction (Fig. 2e). The D-loop conformation in wild-type yeast filaments is inconsistent with the open conformation of the D-loop observed in some vertebrate and plant actin filament structures^23,29^.

The region around residue 167 carries three substitutions between residues 166 and 170 (yeast A167 versus vertebrate E167, F169 versus Y169, and S170 versus A170). This region displays relatively high local rmsd values when the actin monomers are globally aligned (Fig. 2a) but align almost perfectly, including side-chain positions, when local alignment is performed (Fig. 2f).

### Approximately half of the A167E yeast mutant actin filaments resemble rabbit actin more than wild-type yeast actin

Unlike the wild-type data set, which only provided a single conformation, the A167E mutant data resulted in two categories (referred to as A167E^WT^ and A167E^RA^), which produced density maps with two distinct D-loop conformations. A167E^WT^ comprises 54% of the data and with a D-loop conformation that resembles the one observed in the wild-type yeast filament. (Fig. 2g, h). The 167 region of the A167E^WT^ model aligns well with both yeast wild type and vertebrate actin (Fig. 2i). A167E^RA^ contains 46% of the data and displays a D-loop conformation reminiscent of the primary conformation observed in rabbit vertebrate actin^22,23^. This conformation notably differs from the D-loop conformation in yeast wild-type actin (Fig. 2j). In particular, the two peptides at G46 and G48 are flipped relative to the wild-type and A167E^WT^ configurations (Fig. 2k, Supplementary Fig. S2). Except for the direction of E167, the 167 region in A167E^RA^ aligns similarly to the 167 region in A167E^WT^ with wild-type actin (Fig. 2l, Supplementary Fig. S3).

The differences between the D-loops in A167E^RA^ and wild-type yeast actin filaments are comparable to those between the rabbit actin and wild-type yeast structures (Fig. 2e, f, k, l). These differences occur within the same region identified in earlier observations of two alternative conformations in vertebrate actin^22^. However, they exhibit significant dissimilarities (Supplementary Fig. S4), underscoring the overall plasticity of this region. The D-loop α-carbon traces between the A167E^RA^ structure and rabbit actin closely resemble each other (Fig. 2m), including the direction of the peptide bonds at residues G46 and G48 (Fig. 2n). However, the side chains of residue M47 do point in opposite directions. The 167 regions of A167E^WT^ and rabbit actin also resemble each other closely, including the E167 side chain (Fig. 2o). In conclusion, it appears that the divergence in the D-loop conformation at residues G46 and G48 is determined primarily by the residue at position 167 (alanine versus glutamic acid) and not by the sequence variation in the D-loop itself (V43 versus I43).

### The introduction of the A167E mutation into yeast actin introduces a negatively charged patch at the subunit interface

**Fig. 3:**
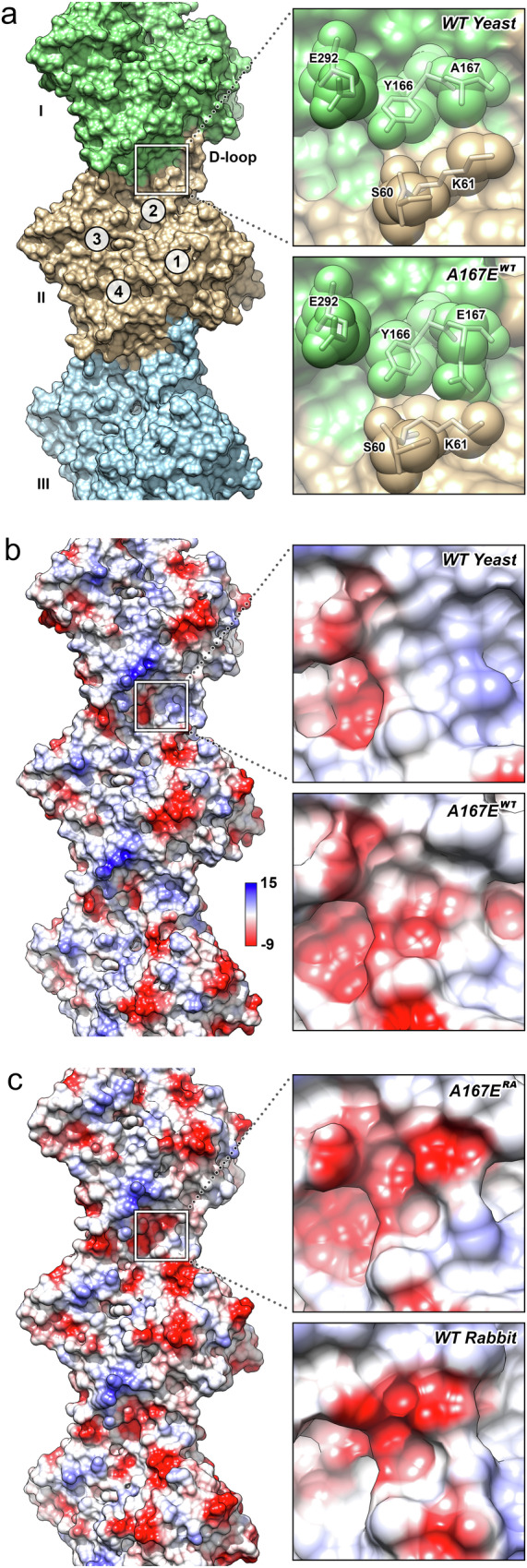
The A167E mutation generates an additional negatively charged patch at the long-pitch subunit interface. **a** Solvent-accessible surface of three subunits of wild-type yeast actin along the long-pitch helix (labeled I–III). The D-loop is indicated for the central subunit II. The upper inset shows the surface at the interface near residue 167, the lower inset shows the same region in yeast mutant A167E^WT^ actin. **b**, **c** Solvent-accessible surfaces of three subunits of wild-type (**b**) and A167E^RA^ mutant (**c**) yeast actin along the long-pitch helix colored with the electrostatic potential of the filament mapped onto the surface. Units are in kcal/(mol *e*) at 298 K. The upper inset shows the surface at the interface near residue 167, the lower inset shows the same region in yeast mutant A167E^WT^ actin. Note that a distinct negatively charged (red) patch appeared if compared to wild-type actin.

A comparison between the electrostatic surfaces of wild-type yeast actin and the A167E^WT^ conformation shows that the substitution leads to the emergence of a negatively charged patch in the vicinity of E167 that bridges the subunit interface, while the rest of the electrostatic surface remains largely unchanged (Fig. 3a–c). The subtle differences between the A167E^WT^ and A167E^RA^ conformations lead to some minor alterations, but there is also a marked rise in the negative charge of the patch (Fig. 3c). The anionic patch spans the inter-subunit interface and involves residues Y166, E167, and E292 of one filament subunit as well as residues S60, and K61 of the adjacent subunit (Fig. 3a).

The presence of E167 in one subunit and K61 in the neighboring subunit allows the formation of a salt bridge, which is not possible in the wild-type yeast structure due to the presence of alanine instead of glutamic acid at position 167. The negative charges of E167 significantly alter the surface charges in its surroundings, leading to the creation of a negatively charged patch at the inter-subunit interface that is poised to bind a cation.

### The negatively charged pocket contains a cation that acts as a bridge between the actin subunits and stabilizes the salt bridge

The negatively charged patch adjacent to E167 presents a plausible binding site for cations. This site is further supported by the fact that E167 conveys cation-dependent stiffness behavior to yeast actin, which positions the cation binding site in the vicinity of residue 167^6,7,11^. However, since the resolution of the A167E^RA^ density map is insufficient to identify the contents of the pocket, we examined this feature in the highest-resolution density map of actin filaments that include Mg^2+^-nucleotide, the rabbit skeletal muscle actin filament in the Mg^2+^ ADP-BeF_3_ state (EMDB code EMD-15104, PDB code 8A2R) at 2.17-Å resolution^12^. We developed an optimized sharpening method to improve the resolution of features in the outer regions of the filament without excessively amplifying noise. This procedure successfully enhanced the definition of features in the periphery where the density peaks were relatively weak and led to marked improvements in the remainder of the structure (Supplementary Fig. S5).

**Fig. 4:**
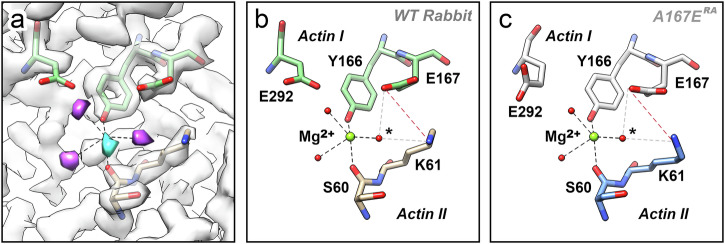
The region near residue E167 in rabbit actin contains Mg^2+^. **a** Sharpened density of rabbit actin (based on EMDB-15104) near residue E167 with un-interpreted peaks labeled in magenta and cyan. The fit of selected residues (extracted from PDB code 8A2R) is also shown. Distances between peaks and modeled atoms that are within the Mg^2+^ coordination distance (<2.6 Å) are indicated as dashed lines. The individual distances are shown in Supplementary Fig. S8. **b** Model after placing an Mg^2+^ ion in the cyan peak and water molecules in the magenta peaks. The water molecule indicated by an asterisk forms hydrogen bonds (gray dashed lines) with residue E167 and residue K61 of the neighboring subunit, stabilizing the salt bridge (dark red dashed line) between the two. In addition, residue Y166 and residue S60 in the neighboring subunit are involved in coordinating the ion, forming a new bridge between the subunits. **c** Because the relative geometry between the residues involved is nearly identical in yeast mutant A167^RA^ actin, all interactions are also possible in this configuration.

Our strategy allowed us to propose the most likely binding site and coordination of a cation at the negatively charged patch. The density map revealed several clear peaks in the pocket that could not be explained by the protein structure alone (Fig. 4a). Similar peaks occur at this site in sharpened high-resolution density maps of vertebrate actin filaments in the Mg^2+^ ADP.Pi state, and to a somewhat lesser extent, in the Mg^2+^ ADP state (Supplementary Fig. S6). The attenuation in peak strength aligns with the observation that Mg^2+^-ADP-BeF_3_ (an Mg^2+^-ATP analog) and Mg^2+^ ADP.Pi actin filaments are stiffer than Mg^2+^-ADP filaments^30^, which suggests that this site is more readily occupied in the former two states. This may indicate that the observed nucleotide-dependent stiffening arises from nucleotide-dependent modulation of cation binding at this site.

By analyzing the distances and angles between these peaks and the surrounding residues using evaluation criteria derived from metal-binding sites of high-resolution structures^31,32^, we were able to pinpoint the peak that most likely represents the bound cation (Fig. 4b). Somewhat surprisingly, the proposed cation does not directly interact with E167, but rather with the hydroxyl group at the tip of the Y166 phenol ring and the side chain oxygen of S60 in the neighboring actin subunit, thereby providing a stabilizing interaction between the two subunits. In addition, the bound cation would anchor a water molecule that stabilizes the salt bridge between E167 and K61 by forming hydrogen bonds with the side chains of both residues. Because the geometry of the A167E^RA^ structure in this region is almost identical to that of the rabbit actin structure, all conclusions derived for potential cation binding at this patch for rabbit actin can be transferred to the A167E^RA^ structure (Fig. 4c).

The buffer of the sample underlying the Mg^2+^ ADP-BeF_3_ reconstruction contains two types of cations, the divalent Mg^2+^ and the monovalent K^+^. The stiffness site binds both monovalent and divalent cations but with different affinities^6^. The affinity for Mg^2+^ is higher than that for monovalent cations, and under the experimental as well as physiological conditions, filament stiffness sites are partially saturated with Mg^2+^ ions^6,7^. Additionally, an analysis of the coordination geometry^31,32^ indicated that Mg^2+^ is a more likely candidate than K^+^ for the peak at the stiffness site. Nevertheless, given that the experimental conditions include K^+^ in the buffer and that Mg^2+^ is not likely to occupy all stiffness sites in a filament, it is conceivable that a certain portion of the stiffness sites within the data may be occupied by K^+^. To clarify the nature of the stiffness site peak, we determined the structure of rabbit vertebrate Mg^2+^ ADP actin with an overall resolution of 2.27 Å resolution (Supplementary Fig. S7, Table 1) under conditions that exclusively include Mg^2+^ ions in the absence of K^+^ ions. Despite the relatively low local resolution, the map replicates the stiffness site peaks observed in the Mg^2+^ ADP-BeF_3_ (Supplementary Fig. S6). Moreover, structures of Ca^2+^ actin filaments, which include K^+^ but not Mg^2+^, fail to replicate the stiffness site peak of the Mg^2+^ ADP-BeF_3_ state despite their extraordinarily high resolution (Supplementary Fig. S6), indicating that the stiffness site is significantly less occupied under those experimental conditions. Collectively, the evidence indicates that Mg^2+^ is the most likely candidate for a bound cation between S60 and Y166.

Coordination numbers five and six are common for Mg^2+^ ions within high-resolution structures of the protein database, with a preference for six^32,33^. Three of the coordinating interactions of Mg^2+^ at this site involve Y166, S60 of the adjacent subunit, and the water molecule stabilizing the salt bridge between E167 and K61. The remaining two coordinating interactions involve two additional water molecules, one potentially forming a hydrogen bond with E292, and the other forming a hydrogen bond with the main-chain oxygen of S60 in the neighboring actin subunit. Together, these interactions form a stable network that strengthens the interactions between the two adjacent actin subunits, thus increasing the filament stiffness. It is possible that the full coordination of the Mg^2+^ ion is octahedral, and the additional interaction involves an unresolved water molecule. While the geometry of the peaks and residues involved in coordination is most consistent with trigonal bipyramid coordination (Supplementary Fig. S8), the resolution of the density map is not sufficient to make a definitive choice.

While the peak arrangement presented here is fully consistent with the placement of an Mg^2+^ ion between residues Y166 and S60 of the neighboring actin subunit, this interpretation is not the only one compatible with the structural data. The coordination of the proposed magnesium ion is quite loose, with the coordination distances mostly at the larger end of the spectrum. In addition, the peak is weaker than typically expected for well-defined bound ions, even though this is consistent with the low observed occupancy and its location near the periphery of the filament. An alternative assignment as a coordinated water molecule is possible and generally compatible with the structural data. However, the demonstrated salt-dependent stiffness behavior of vertebrate and A167E yeast actin^6^ necessitates the presence of a cation near residue E167. The location we assigned is the only one for cation placement in this region that aligns with the structural data. Thus, despite some ambiguity in peak assignment using structural data alone, the biophysical evidence strongly supports our interpretation.

## Discussion

The difference in the D-loop orientations between filamentous wild-type yeast actin and vertebrate actin is mimicked in the two A167E yeast mutant conformations we detected (Fig. 2). The most likely explanation for this behavior is that this conformational change is primarily controlled by the presence of the E167 side chain, which enables the binding of magnesium cations at the stiffness site^6,7^. This event impacts the orientation and stability of the D-loop, most likely via the cross-subunit salt bridge between E167 and K61, the latter of which is located at the subdomain-2 helix that anchors the end of the D-loop (residues 38–52). Another residue potentially contributing to the positioning of the D-loop is F169 (Y169 in vertebrate actin), which is directly coupled to E167 through G168 and inserts into the cavity created by the D-loop of the neighboring actin subunit.

It is plausible that this allosteric effect on the D-loop contributes to the overall stiffness of the filament in addition to the effects of the salt bridge and the inter-subunit interactions of the Mg^2+^ cation. This is consistent with low-resolution density maps of A167E yeast mutants in the presence of high Mg^2+^ and low Mg^2+^ concentrations^11^. The low Mg^2+^-concentration density map shows significantly less density for the D-loop than the high Mg^2+^-concentration structure. The altered orientation and stability of the D-loop are also likely to impact the filament binding of those actin-binding proteins that bind to the D-loop and may contribute to Mg^2+^ dependence of those binding events in the absence of direct interactions with the Mg^2+^ at the stiffness site.

The similarity of the difference in the orientation of the D-loop between wild-type yeast actin and vertebrate actin filaments and that between the two A167E yeast mutant conformations, indicates that the presence of the E167 side chain alone is insufficient to shift the D-loop position to that observed in rabbit actin, and that binding of Mg^2+^ at the stiffness site is a necessary factor. Moreover, the stiffness of the A167E mutant depends on the Mg^2+^ concentration^6^. Together, this suggests that the A167E^RA^ and rabbit actin conformations correspond to the conformation prevalent when Mg^2+^ is bound, while the A167E^WT^ conformation represents the conformation in the absence of Mg^2+^ because the orientation of E167 in this conformation does not support Mg^2+^ binding. Consequently, approximately half of the subunits in the A167E mutant are likely to have Mg^2+^ bound at the stiffness site, while the other half remains unoccupied, consistent with previous results^6,7^.

Many high-resolution actin filament structures show a salt bridge between E167 and K61 of the neighboring subunit, but yeast actin carries an alanine at that position and thus cannot form this salt bridge. The absence of a stabilizing salt bridge in wild-type yeast actin likely contributes to the lower bending stiffness of wild-type yeast actin compared to rabbit skeletal actin. In humans, the K61N mutation has been linked to cancer^34^, highlighting the importance of this region and potentially linking stiffness regulation to human disease. Introducing glutamic acid via the A167E mutation does allow for the formation of this salt bridge, but the resulting mutant filaments are less stiff than wild-type yeast actin at low Mg^2+^ concentrations^11^. Only with an increase in Mg^2+^ concentration does the A167E mutant get stiffer, while the wild type remains the same.

These findings confirm that the salt bridge formed between E167 and K61 alone is not sufficient to explain the bending stiffness of actin filaments^11^. E167 must also enable the binding of cations to a stiffness site on actin filaments, leading to the subsequent increase in stiffness. We observed no direct interaction between the potential cation and E167, contrary to previous predictions based on structural bioinformatics and molecular dynamics simulations using low-resolution structures of actin^6,35,36^. Rather, the potential cation binding alters the actin conformation in a manner that promotes the formation of the ionic bridge between K61 and E167^11^. By examining the high-resolution structures, we found that a water molecule, coordinated by the potential cation and the two salt-bridge residues, stabilizes both the salt bridge and Mg^2+^ binding. If E167 is absent as in yeast wild-type actin, this water molecule will probably be destabilized, which would then lead to the destabilization of Mg^2+^ binding at the proposed stiffness site. In addition to the destabilization of the salt bridge, the absence of the water molecule would then result in a weakening of the inter-subunit contact between Y166 and S60 provided by the proposed cation. In humans, the Y166S mutation in α-cardiac actin has been linked to hypertrophic cardiomyopathy^37^, possibly linking the disease to defects in cation-dependent actin filament stiffness.

Filament bending induces rearrangements in the actin subunits, including the region near the stiffness site^14^. Both inside and outside the bending curve, the change in distance between Y166 and S60 remains below 1 Å (0.65 ± 0.25 Å) with respect to the high-resolution structures of straight filaments^12^, regardless of the nucleotide. This suggests that Y166 and S60 could still contribute to Mg^2+^ coordination in the bent state. However, the distance between E167 and K61 significantly increases outside the bending curve. This change is nucleotide-dependent, measuring 5.8 Å for bent ADP.Pi filaments and 7.8 Å for bent ADP filaments. These distances indicate a break in the interaction between the two residues on the outside of the bending curve. In contrast, the distance between E167 and K61 at the inside of the bending curve shows little change (0.10 ± 0.42 Å), suggesting that the overall geometry of the proposed stiffness site is maintained on the inside of the bending curve of bent filaments. The binding site of vertebrate cofilin with filamentous actin (PDB code 6VAO) does not directly overlap with the position of the proposed Mg^2+^. However, cofilin binding does cause significant perturbations to the stiffness site, particularly by disrupting the inter-subunit salt bridge between E167 and K61. As a result, the Mg^2+^ cation is likely to dissociate upon cofilin binding, explaining the strong thermodynamic coupling between cofilin binding and cation dissociation^11,38^.

The D-loop region of the high-resolution rabbit skeletal muscle density maps has poor definition, with several residues in the region being unresolved^12^. In contrast, in lower-resolution actin filament density maps^23,27,28^, including the yeast actin maps presented here, the backbone of these residues can be confidently modeled. This suggests that the D-loop is variable at the highest resolution but exhibits a fairly narrow distribution of conformational states that can be captured at a somewhat lower resolution. It is interesting to note that stiffening of the filament in A167E yeast mutants does not result in a narrower distribution of D-loop conformations at this resolution but in a shift of the α-carbon trace, potentiating tightening the interactions between the D-loop and the hydrophobic cleft of the adjacent actin subunit.

## Methods

### Yeast actin sample preparation

Yeast wild-type and A167E-mutant actin were purified as described^11^. Briefly, G-actin was purified using DNase-I affinity chromatography, where the protein was eluted from the affinity column with a G-buffer containing 20–25% sucrose, 50% formamide, 5 mM Tris (pH 7.8; 22 °C), 0.2 mM CaCl_2_, 0.3 mM ATP, 1 mM DTT, and 0.2 mM PMSF, and was then applied directly to a DEAE column. Actin was eluted from the DEAE column in G-buffer [10 mM Tris (pH 7.8; 22 °C), 0.2 mM CaCl_2_, 0.2 mM ATP, 1 mM DTT, and 0.2 mM PMSF] containing 350 mM KCl. Sample screening included polymerization of yeast actin samples one or 24 h before vitrification. Ca-ATP-G-actin was converted into Mg-ATP-G-actin with 200 µM EGTA and MgCl_2_ equal to the [G-actin] plus 10 µM. Mg-ATP-G-actin was polymerized by adding 0.1 volume of 10× KMEI buffer (50 mM KCI, 2 mM MgCl_2_, 0.2 mM ATP, 2 mM DTT, 0.2 mM EGTA, and 10 mM imidazole [pH 7.0; 22 °C]) to yield a concentration of ~4 μM filamentous actin of both WT and mutant actin. The mentioned buffer components are in the final concentrations after mixing. Half of the sample was labeled with rhodamine-phalloidin and visualized with a Mic digital objective-based TIRF microscope (Till Photonics) equipped with a 100× oil objective (Olympus), an iXon 897 EMCCD camera (Andor Technology), and Live Acquisition image software (Till Photonics), verifying homogeneity of filament morphology and lack of bundling or aggregates. Screening for the best sample concentration and blotting conditions was performed on a T12 Tecnai Spirit electron microscope (ThermoFisher Scientific) equipped with a 4Kx4K Eagle camera (ThermoFisher Scientific), operated at a voltage of 120 kV. Polymerized samples were diluted into KMEI buffer at 0.125 mg/ml actin. In total, ~5 μL was applied to plasma-cleaned C-flat copper grids 2/1 or 2/2 (Protochips Inc.), respectively. After 1 minute of incubation in a humidified chamber, excess liquid was manually blotted, and the samples were plunge-frozen in liquid nitrogen-cooled liquefied ethane using an in-house designed cryo-plunger. The 24-h polymerized samples reproducibly provided homogenous and consistent sample loading and hence were used for data acquisition.

### Sample preparation of rabbit actin in the absence of K^+^

Rabbit skeletal muscle actin was purified as previously described^39^. Briefly, actin was purified from back and leg muscles by gel-filtering over Sephacryl S-300 equilibrated in buffer A (2 mM Tris pH 8, 0.2 mM CaCl_2_, 1 mM NaN_3_, 0.2 mM ATP, and 0.5 mM dithiothreitol [DTT]). Right before polymerization, actin monomers with bound Ca^2+^ were converted to Mg^2+^ actin by equilibrating with MgCl_2_ with a concentration equal to the actin concentration plus an extra 10 μM and 0.2 mM EGTA (pH 7.5) for 5 min on ice with occasional tapping to remix. Rabbit actin was polymerized by adding 10× polymerization buffer to final concentrations of 10 mM imidazole pH 7.5, 1 mM MgCl_2_, 2 mM DTT, and 0.2 mM ATP, incubated at room temperature for 1 h. The polymerized actin was kept on ice. Note that the polymerization buffer did not contain KCl. Two separate samples with different Mg^2+^ cation concentrations were examined to rule out the sample preparation dependence of peaks near the stiffness site. For the first sample, about 1 min before freezing, the polymerized actin was mixed 1:1 with a solution of 10 mM imidazole pH 7.5, 1 mM DTT, and 1.2 mM EDTA. The final Mg^2+^ in solution is ~0.5 mM. For the second sample, the polymerized actin was mixed 1:5 with a solution of 10 mM imidazole pH 7.5, 1 mM DTT, and 11.8 mM MgCl_2_, and the final Mg^2+^ in the solution is 10 mM. The samples were frozen on glow-discharge-treated Quantifoil 1.2/1.3 300-mesh (Holey carbon) grids. A sample of 3.0 μL was applied onto the carbon side of the grid using a Vitrobot Mark IV (Thermo Fisher Scientific) at 4 °C and 100% humidity. The samples were plunge-frozen into liquid ethane at liquid nitrogen temperature.

### Cryogenic electron microscopy data acquisition

The yeast actin datasets for structure determination were acquired on a Titan Krios electron microscope (ThermoFisher Scientific) equipped with an XFEG and operated at a voltage of 300 kV. Although the sample preparation protocol was optimized, we had to screen for usable grids and grid squares manually. Images were recorded with Falcon 2 (ThermoFisher Scientific) direct detection device under minimal dose conditions using the automatic data collection software EPU (ThermoFisher Scientific). Within each selected grid hole, two positions were imaged, each with a total exposure of 1 s. A total of 1051 wild-type and 1286 A167E-mutant actin dose-fractionated image stacks with seven frames each were collected with a 1.035-Å pixel size at defoci ranging from −0.8 µm to −2.8 µm. The rabbit actin data sets for image reconstructions were collected using a Titan Krios equipped with an X-cold field emission gun at 300 kV utilizing a Gatan image filter with a slit width of 20 eV in nanoprobe mode. Defocus values between −1.2 μm and −2.5 μm were applied. A K3 Gatan summit camera in super-resolution mode was used to collect one dose-fractionated image stack per hole using serialEM^40^ data collection software. Each stack contained 35 frames with a frame time of 0.04 s. A dose rate of 26 counts/pixel/s and a physical pixel size of 0.829 Å was used. In total, 4392 image stacks were collected for the first sample preparation condition and 3851 for the second.

### Image processing and structure determination of yeast actin data

Dose weighting and motion correction were applied using MotionCor2^41^ with 5 × 5 patches. The defocus was estimated using CTFFIND4^42^. The motion-corrected images were processed with the helical reconstruction routines in RELION version 3.08^43^. Primarily long straight filaments were manually selected within RELION. For the wild-type yeast actin data set, a total of 416,447 filament segments for the A167E-mutant, and 742,393 filament segments were extracted using a box size of 200 × 200 pixels and an inter-box distance of 33 pixels. Next, two-dimensional reference-free classification for each data set was carried out in RELION to eliminate bad segments, reducing the number of segments to 338,175 and 582,685 segments, respectively. The goal of the processing strategy described below was to separate possibly subtle conformations triggered by the presence of cations at the stiffness site. To that end, we employed a multi-reference strategy that was inspired by the ISAC procedure^44^, and aims to obtain stable 3D classes through splitting, iterative 3D classification, class evaluation, regrouping of segments, and updating of references (Supplementary Fig. 9). All steps of this protocol were run using the subset selection and 3D refinement features of the RELION graphical user interface. The procedure was initiated by splitting the initial set of segments into eight random subsets. Each of these subsets was subjected to unsupervised 3D classification in RELION with three classes (*k* = 3) and a synthetic helix made of soft spheres and vertebrate actin’s helical parameters (27.5 Å rise, −166.66° twist) as a starting model. Next, the initial set of segments was separated into three groups according to the similarity between the classification results of the eight subsets. The references for each of the three groups were updated individually, then low-pass filtered to 35 Å to serve as a reference for the next iteration. Each of the three groups was then split into eight random subsets, each of which was subjected to unsupervised 3D classification (*k* = 3). The segment set was then regrouped into three sets according to similarities of the new classification results, the references were updated individually, and another iteration was performed. After three such iterations, the regrouping appeared stable without further changes, and segments were compiled into one (wild-type) or two (A167E mutant) groups according to the final classification results. Segments that contributed to low-resolution or distorted classes were discarded. Each of the final groups was subjected to 3D auto-refinement with helical symmetry and a Z-mask of 90%, followed by post-processing with a Z-mask of 60%, both in RELION. Postprocessing consisted of B-factor sharpening and the application of a soft-edged mask. The estimated resolution of the resulting yeast wild-type reconstruction, using the 0.143 Fourier-shell-correlation (FSC) cutoff gold-standard procedure implemented in RELION, reached a resolution of 4.43 Å with a total of 238,930 contributing segments. The A167E mutant data converged to two separate reconstructions at 3.83-Å resolution with 195,534 contributing segments (A167E^RA^) and 3.98-Å resolution with 170,753 contributing segments (A167E^WT^). After fitted models were available, an optimal sharpening procedure (see below) using pyCoAn 0.4 (github.com/pycoan/distro) an extended Python version of CoAn^45^ to further improve the interpretability of the maps. Local resolution estimates were calculated with RELION.

### Image processing and structure determination of rabbit actin data

The datasets of the two sample preparation conditions were processed separately. Micrographs were subjected to motion correction using MotionCor2^41^ and CTF estimation using Gctf^46^ before particle picking using michelixtrace from the SPRING package^47^. Subsequent steps were carried out in CryoSPARC^48^ version 2. In total, 2,374,404 and 3,650,568 helical segments were extracted from micrographs for the two conditions, respectively. The picked segments were then subjected to 2D classification to remove spurious particles, yielding a total of 2,291,417 and 3,628,800 segments. These segments were then subjected to 3D structure refinement using the “Helical refinement” job in CryoSPARC. This step was followed by local and global CTF refinement, yielding final resolutions of the reconstructions of 2.45 Å for the first condition and 2.29 Å for the second condition. Examination of the reconstructions did not reveal any discernible differences, and the data was merged, yielding a combined reconstruction of 2.27 Å resolution.

### Structural modeling and refinement

The 4.1-Å resolution model of actin skeletal muscle actin in the Mg^2+^ ADP state (PDB code 5ONV)^23^ was either used directly for the rabbit actin reconstruction or altered to the yeast sequence with the aid of ChimeraX 1.3^49^. The models were fitted into each of the reconstructions via interactive, molecular-dynamics-assisted fitting of a single monomer using Isolde 1.4^50^. Following this step, an Isolde refinement of five subunits was performed to address energy strains at the interfaces. As a final step, Phenix 1.20.1^51^ was used to conduct an automated real-space refinement with three macrocycles, incorporating global minimization, secondary structure constraints, symmetry constraints, and one round of temperature-factor refinement after completion of the three macrocycles. Quality indicators, including MolProbity^52^ and EMRinger^53^ scores, were calculated with Phenix. Q-scores^54^ were calculated with Chimera^55^. The analysis of distance changes upon bending was conducted using atomic models of bent Mg^2+^ ADP (PDB code 8D15) and bent Mg^2+^ ADP.Pi (PDB code 8D16) filaments^14^. The analysis of the stiffness site geometry was conducted with the CMM server^31^. The nVECSUM parameter^31^, which measures the consistency with the ideal metal-ligand bond distances (larger is more consistent), is 0.19 for Mg^2+^ and 0.18 for K^+^, the gRMSD parameter^31^, measuring the deviation of all angles from idealized coordination geometry (smaller is better) is 14.2° for Mg^2+^ and 15.9° for K^+^. For reference, these values were 0.2 and 6.2° for the Mg^2+^ at the nucleotide site. Figures were generated with Chimera and ChimeraX.

### Optimized sharpening procedure

Optimized sharpening was based on maximizing the EMRinger score as a function of sharpening parameters. For this purpose, we implemented real-space sharpening in pyCoAn. The algorithm calculates a low-pass filtered version of the original density with a fixed kernel of 3x3x3 pixels and subtracts a weighted version of the low-pass-filtered density from the original. The adjustable parameter for this filter is the weight (sharpening factor). Note that this procedure is not equivalent to B-factor sharpening. The filter is essentially a 3D version of high-boost filtering where high-frequency components are enhanced without eliminating low-frequency information. For each trial sharpening factor, a filtered density map was calculated. Next, the model refined into the original density was subjected to one cycle of real-space refinement in Phenix with the sharpened map to allow for minor adjustments. The EMRinger score was calculated and recorded. This process was repeated for several sharpening factors until the EMRinger score maximum was identified. This optimized sharpening procedure was applied to all three yeast actin reconstructions and the published 2.17-Å resolution rabbit actin density (EMD-15104). All other maps, including the 2.27-Å rabbit skeletal actin map derived in this study, were subjected to a modified sharpening procedure that skipped the EMRinger scoring and, instead, optimized the appearance of the density at residue Y166 with main emphasis on the appearance of the hole in the aromatic ring.

### Reporting summary

Further information on research design is available in the Nature Portfolio Reporting Summary linked to this article.

### Supplementary information

Supplementary Material

Description of Additional Supplementary Files

Supplementary Data 1

Supplementary Data 2

Supplementary Data 3

Supplementary Data 4

Reporting Summary

## Data Availability

Density maps are deposited at the EMDB (accession codes EMD-41273, EMD-41274, EMD-41279, EMD-43763), and atomic models at the PDB (accession codes 8THX, 8THY, 8TI3, 8W36). The validation reports are provided as Supplementary Data 1–4. All other data supporting the findings of this study are available within the paper and its Supplementary Information Files.
