# Supplementary material for: High-resolution yeast actin structures indicate the molecular mechanism of actin filament stiffening by cations
Source: Commun Chem. 2024 Jul 30;7:164. doi: 10.1038/s42004-024-01243-x (PMC11289367; doi:10.1038/s42004-024-01243-x)
Supplement: Supplementary file 1 — Supplementary Material [file 42004_2024_1243_MOESM1_ESM.pdf]

High-resolution yeast actin structures indicate the molecular mechanism of actin filament stiffening by cations

Xiao-Ping Xu[1], Wenxiang Cao[2], Mark F. Swift[1], Nandan G. Pandit[2], Andrew E. Huehn[2], Charles V. Sindelar[2], Enrique M. De La Cruz[2], Dorit Hanein*[,3], Niels Volkmann*[,4]

Supplementary Material:

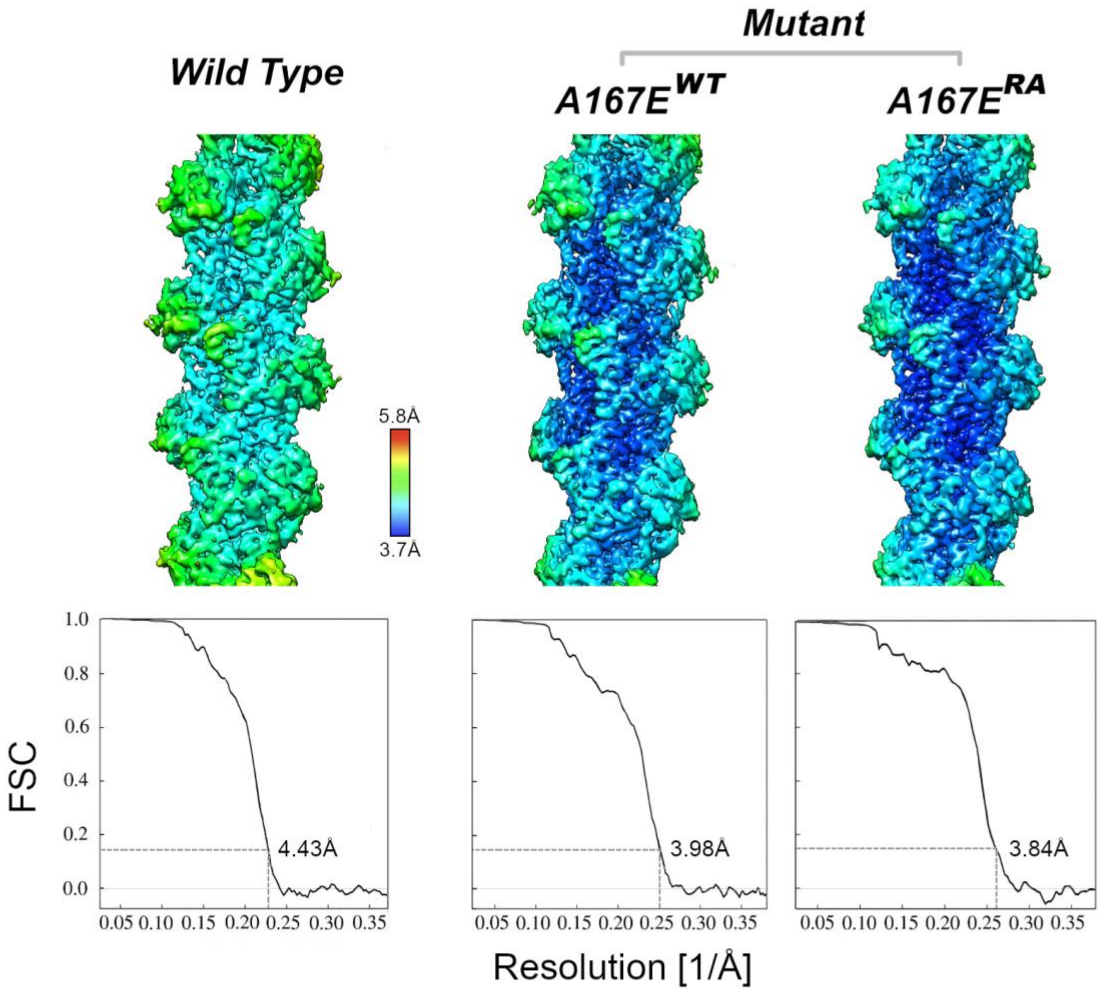

Supplementary Figure S1: ***Local resolution of the three yeast actin reconstructions.*** Fourier Shell Correlation (FSC) curves are shown below the reconstructions. The 0.143 criterion was used to estimate the overall resolution, which is indicated inside the curve windows.

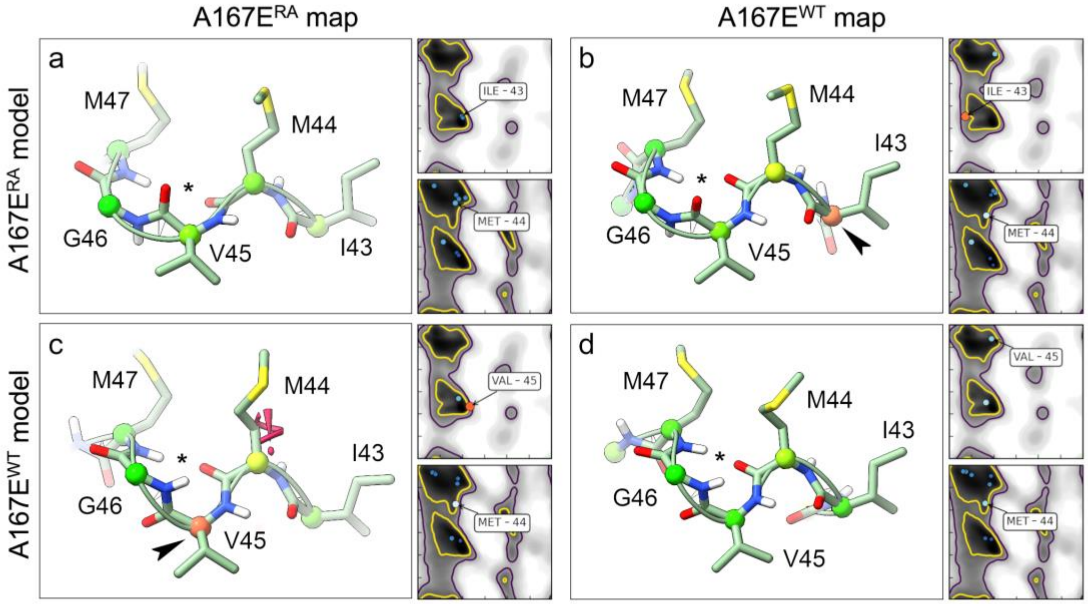

Supplementary Figure S2: ***Comparison of A167E^{RA} and A167E^{WT} models in the D-loop region.*** Ramachandran plot compatibility is mapped onto the α-carbons (green: inside the expected region, red: outliers). Ramachandran plots with highlighted residues are shown as insets to the right of each panel. If the probability of the rotamer geometry drops, below reasonable levels, a symbol is drawn next to the residue. The severity of the probability drop is proportional to the color going from yellow (moderate) to red (severe). If either of the models is allowed to refine using the density and molecular dynamics force field without allowing changes of the stereochemistry into the matching map, all sidechain and main chain geometries are excellent (a and d). If either of the models is allowed to refine into the opposite density, the geometry gets distorted (b, c). Violations occur at I43 when the A167E^{RA} is refined into the A167E^{WT} map (b) and M44 V45 (arrowhead) and M44 (rotamer symbol) when the A167E^{WT} is refined into the A167E^{RA} map (c).

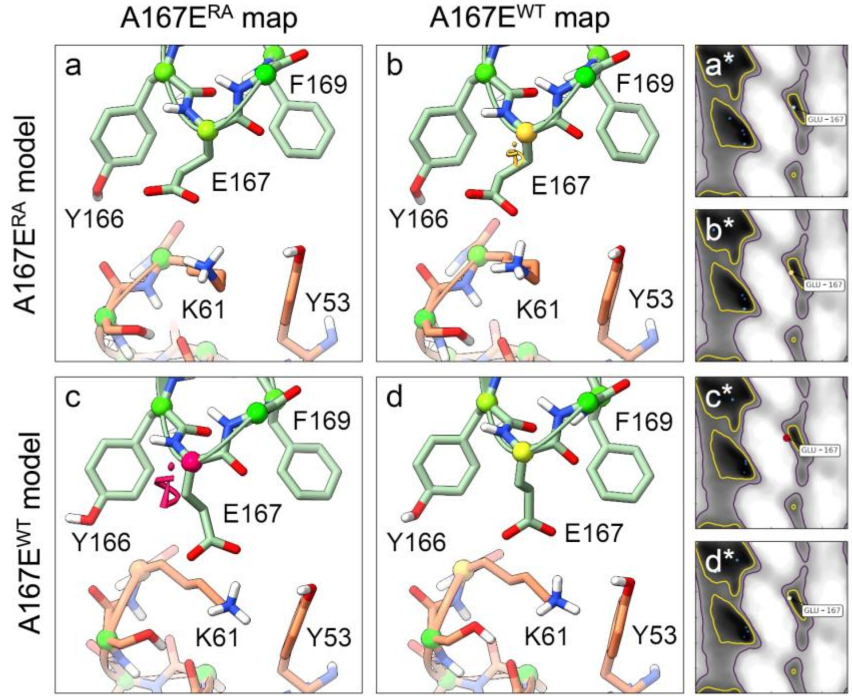

Supplementary Figure S3: ***Comparison of A167E^{RA} and A167E^{WT} models near the stiffness site.*** Ramachandran plot and the probability of the rotamer are indicated as described in Extended Data Figure 2. If either of the models is allowed to refine with density and force-field constrains only into the matching map, all sidechain and main chain geometries are within the expected bounds (a and d). If either of the models is allowed to refine into the opposite density, the geometry gets distorted (b, c). Severe violations to both the Ramachandran plot and rotamer probability parameters occur at E167 when the A167E^{RA} is refined into the A167E^{WT} map (b). When the A167E^{WT} is refined into the A167E^{RA} map, violations occur to a somewhat lesser but still noticeable extent (c).

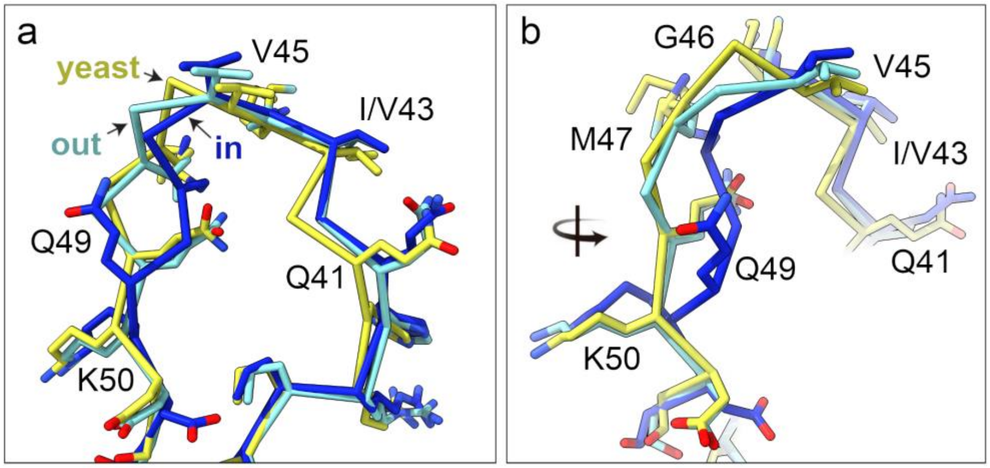

Supplementary Figure S4: ***D-loop differences.*** The differences observed between two alternative D-loop conformations in chicken vertebrate actin ("in" versus "out") are dissimilar to the difference between the A167E^{RA} conformation (which closely matches the "out" conformation) and A167E^{WT} conformation of yeast actin (yellow).

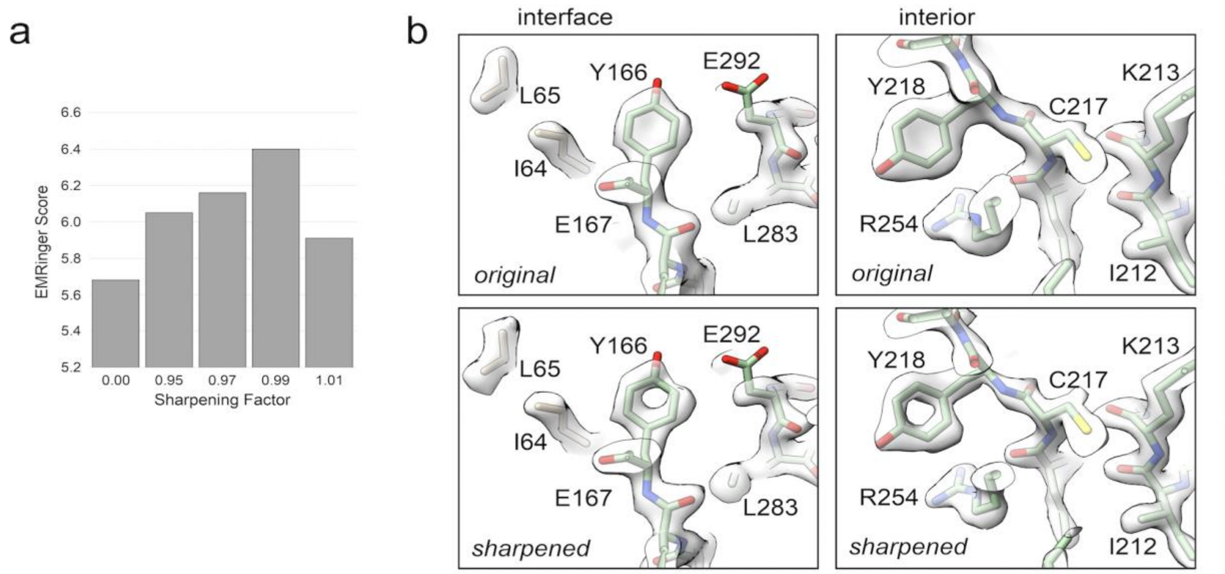

Supplementary Figure S5: ***Optimized sharpening of density map.*** **(a)** Dependence of EMRinger score on sharpening factor. **(b)** Improvement of density after sharpening with the optimized sharpening factor (0.99). Two representative regions, one at the interface, and one in the interior of the molecule are shown. With the sharpening, the match between density and model gets generally tighter and holes in aromatic rings start to appear in both regions

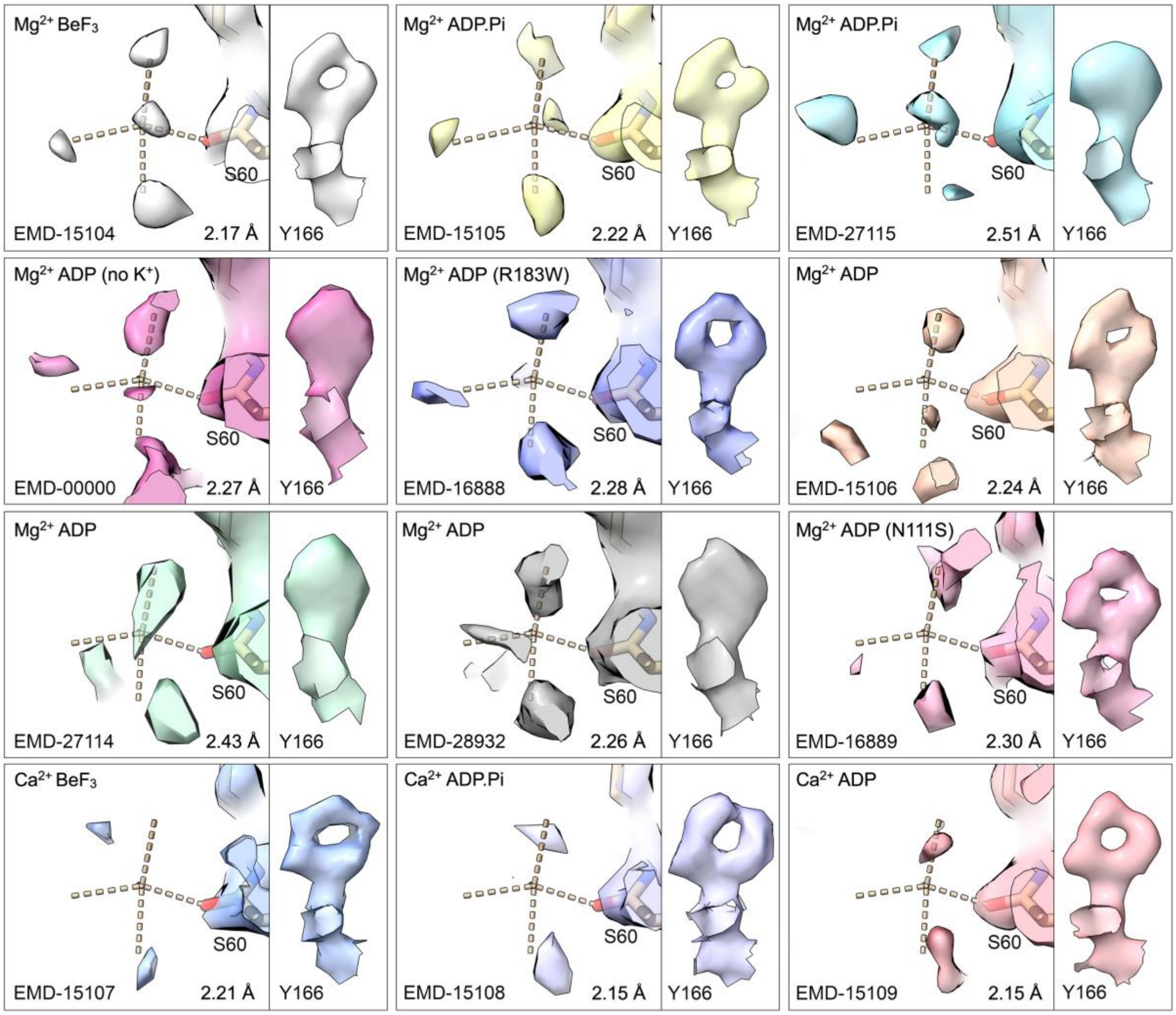

Supplementary Figure S6: ***Analysis of peaks near the stiffness site in various high-resolution actin filament densities after local sharpening.*** The nucleotide is indicated in the left upper corner of each main panel, the EMDB accession code in the lower left corner, and the overall resolution in the right lower corner. The view of the main panel is along the connection towards Y166. The geometry of the Mg^{2+} coordination determined from the Mg^{2+} ADP BeF_3 map (dashed lines) is shown for reference in each panel. Only peaks within 1.5 Å of the originally assigned peaks and density associated with S60 are shown for clarity. On the right of each panel, the density for residue Y166 is shown as an indicator of the local resolution in the region after local sharpening.

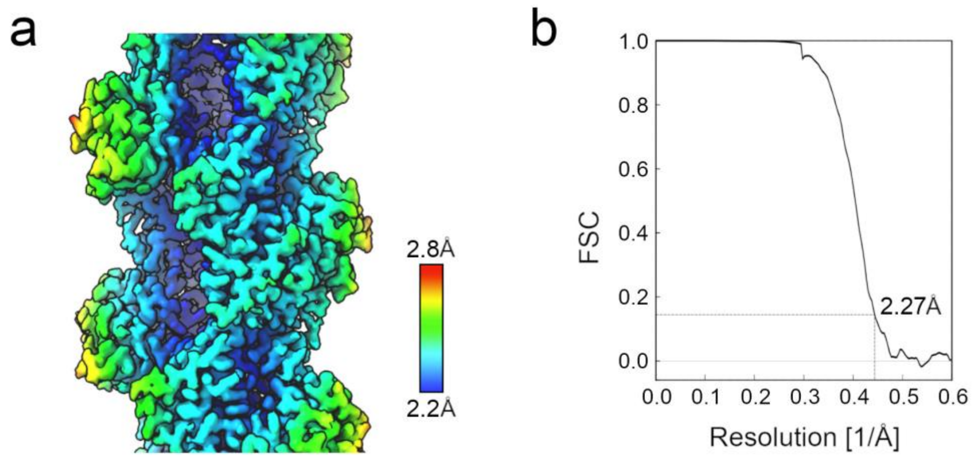

Supplementary Figure S7: Mg^{2+} ADP *Rabbit skeletal actin reconstruction in the absence of K^{+}.* a. Local resolution. **b.** Fourier Shell Correlation (FSC). The 0.143 criterion was used to estimate the overall resolution, indicated by a dashed line.

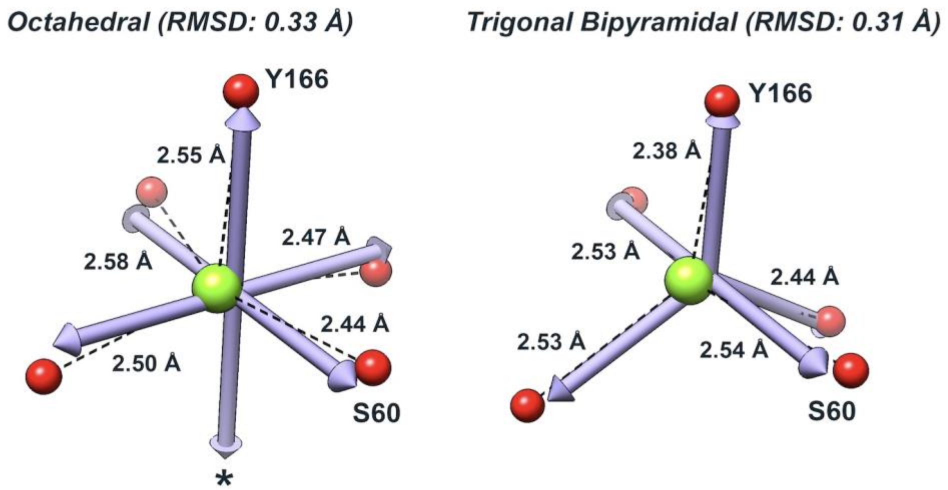

Supplementary Figure S8: ***Potential coordination of Mg^{2+} ion in the wild-type rabbit structure.*** Both octahedral and trigonal bipyramidal arrangements are consistent with the experimental data. The figure shows the optimal arrangements for both coordination types within the data constraints. One water molecule necessary for octahedral coordination is not resolved in the density (asterisk).

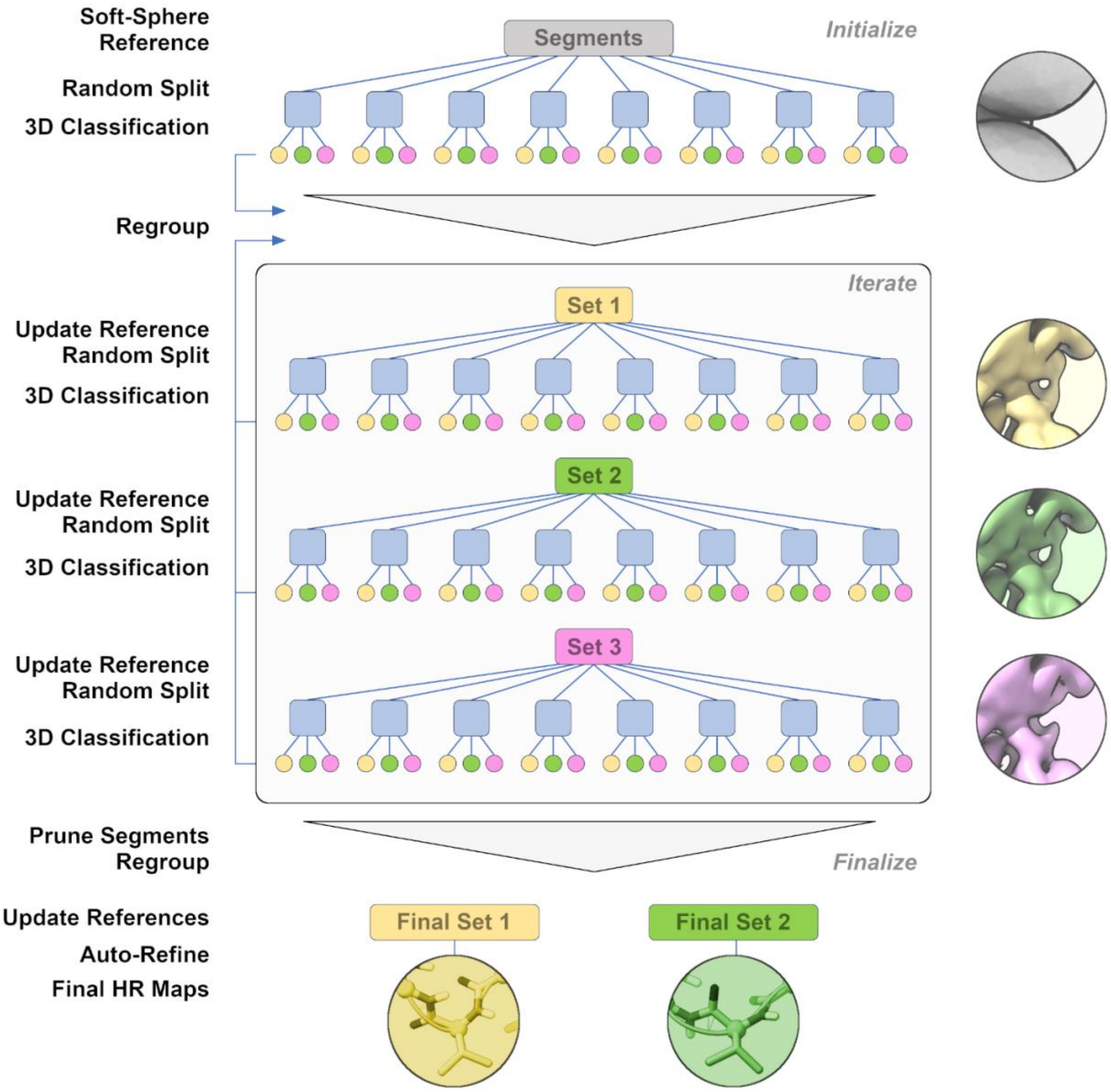

Supplementary Figure S9: ***Depiction of processing workflow employed for the analysis of wild-type and A167A mutant yeast actin data sets.*** On the right, the D-loop region of the starting model (top, grey) and typical 3D classification results are shown (yellow, green, pink). Despite the low resolution, differences are readily identifiable and can be used to regroup the segments based on which map they contribute to. Before the next iteration, these densities are low-pass filtered and used as references for the respective segment groups. For the wild-type data, two of the three classes became more and more similar through the iterations until they were not distinguishable anymore. For the A167E data set the differences became more distinct and stabilized in appearance after three iterations. The pictures near the "Final Set" boxes show the differences in the vicinity of residues V45 and G45 for the two mutant map conformations.
