# Supplementary material for: High-resolution yeast actin structures indicate the molecular mechanism of actin filament stiffening by cations
Source: Commun Chem. 2024 Jul 30;7:164. doi: 10.1038/s42004-024-01243-x (PMC11289367; doi:10.1038/s42004-024-01243-x)
Supplement: Supplementary file 2 — Description of Additional Supplementary Files [file 42004_2024_1243_MOESM2_ESM.pdf]

Description of Additional Supplementary Files

File name- Supplementary Data 1-4

File description- The validation reports are provided as SUPPLEMENTARY DATA 1-4
