# Supplementary material for: High-resolution yeast actin structures indicate the molecular mechanism of actin filament stiffening by cations
Source: Commun Chem. 2024 Jul 30;7:164. doi: 10.1038/s42004-024-01243-x (PMC11289367; doi:10.1038/s42004-024-01243-x)
Supplement: Supplementary file 4 — Supplementary Data 2 [file 42004_2024_1243_MOESM4_ESM.pdf]

Full wwPDB EM Validation Report (i)

Jul 19, 2023 – 10:00 AM EDT

PDB ID	:	8THY
EMDB ID	:	EMD-41274
Title	:	yeast actin A167E mutant rabbit-like
Deposited on	:	2023-07-18
Resolution	:	3.84 Å(reported)

This wwPDB validation report is for manuscript review

For Manuscript Review

This is a Full wwPDB EM Validation Report.

This report is produced by the wwPDB biocuration pipeline after annotation of the structure.

We welcome your comments at *validation@mail.wwpdb.org*
A user guide is available at
https://www.wwpdb.org/validation/2017/EMValidationReportHelp
with specific help available everywhere you see the (i) symbol.

The types of validation reports are described at
http://www.wwpdb.org/validation/2017/FAQs#types.

The following versions of software and data (see references (i)) were used in the production of this report:

MolProbity	:	4.02b-467
Mogul	:	1.8.5 (274361), CSD as541be (2020)
buster-report	:	1.1.7 (2018)
Percentile statistics	:	20191225.v01 (using entries in the PDB archive December 25th 2019)
Ideal geometry (proteins)	:	Engh & Huber (2001)
Ideal geometry (DNA, RNA)	:	Parkinson et al. (1996)
Validation Pipeline (wwPDB-VP)	:	2.34

1 Overall quality at a glance (i)

The following experimental techniques were used to determine the structure:
ELECTRON MICROSCOPY

The reported resolution of this entry is 3.84 Å.

Percentile scores (ranging between 0-100) for global validation metrics of the entry are shown in the following graphic. The table shows the number of entries on which the scores are based.

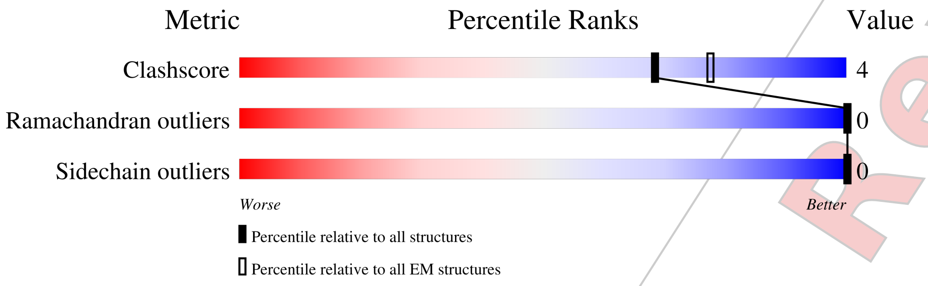

Metric	Whole archive (#Entries)	EM structures (#Entries)
Clashscore	158937	4297
Ramachandran outliers	154571	4023
Sidechain outliers	154315	3826

The table below summarises the geometric issues observed across the polymeric chains and their fit to the map. The red, orange, yellow and green segments of the bar indicate the fraction of residues that contain outliers for >=3, 2, 1 and 0 types of geometric quality criteria respectively. A grey segment represents the fraction of residues that are not modelled. The numeric value for each fraction is indicated below the corresponding segment, with a dot representing fractions <=5%

Mol	Chain	Length	Quality of chain
1	A	370	92% 8%
1	B	370	93% 7%
1	C	370	91% 9%
1	D	370	93% 7%
1	E	370	94% 6%

For Manuscript Review

2 Entry composition ⓘ

There are 3 unique types of molecules in this entry. The entry contains 14600 atoms, of which 0 are hydrogens and 0 are deuteriums.

In the tables below, the AltConf column contains the number of residues with at least one atom in alternate conformation and the Trace column contains the number of residues modelled with at most 2 atoms.

- Molecule 1 is a protein called Actin.

Mol	Chain	Residues	Atoms					AltConf	Trace
1	A	370	Total 2892	C 1833	N 489	O 551	S 19	0	0
1	B	370	Total 2892	C 1833	N 489	O 551	S 19	0	0
1	C	370	Total 2892	C 1833	N 489	O 551	S 19	0	0
1	D	370	Total 2892	C 1833	N 489	O 551	S 19	0	0
1	E	370	Total 2892	C 1833	N 489	O 551	S 19	0	0

There are 5 discrepancies between the modelled and reference sequences:

Chain	Residue	Modelled	Actual	Comment	Reference
A	167	GLU	ALA	variant	UNP P60009
B	167	GLU	ALA	variant	UNP P60009
C	167	GLU	ALA	variant	UNP P60009
D	167	GLU	ALA	variant	UNP P60009
E	167	GLU	ALA	variant	UNP P60009

- Molecule 2 is ADENOSINE-5'-DIPHOSPHATE (three-letter code: ADP) (formula: $C_{10}H_{15}N_5O_{10}P_2$) (labeled as "Ligand of Interest" by depositor).

For Manuscript Review

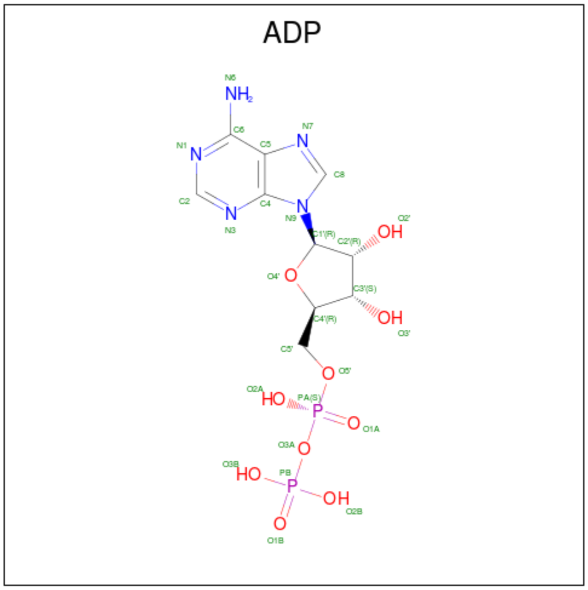

Mol	Chain	Residues	Atoms	AltConf
2	A	1	Total C N O P<br>27 10 5 10 2	0
2	B	1	Total C N O P<br>27 10 5 10 2	0
2	C	1	Total C N O P<br>27 10 5 10 2	0
2	D	1	Total C N O P<br>27 10 5 10 2	0
2	E	1	Total C N O P<br>27 10 5 10 2	0

- Molecule 3 is MAGNESIUM ION (three-letter code: MG) (formula: Mg) (labeled as "Ligand of Interest" by depositor).

Mol	Chain	Residues	Atoms	AltConf
3	A	1	Total Mg<br>1 1	0
3	B	1	Total Mg<br>1 1	0
3	C	1	Total Mg<br>1 1	0
3	D	1	Total Mg<br>1 1	0
3	E	1	Total Mg<br>1 1	0

For Manuscript Review

3 Residue-property plots ⓘ

These plots are drawn for all protein, RNA, DNA and oligosaccharide chains in the entry. The first graphic for a chain summarises the proportions of the various outlier classes displayed in the second graphic. The second graphic shows the sequence view annotated by issues in geometry. Residues are color-coded according to the number of geometric quality criteria for which they contain at least one outlier: green = 0, yellow = 1, orange = 2 and red = 3 or more. Stretches of 2 or more consecutive residues without any outlier are shown as a green connector. Residues present in the sample, but not in the model, are shown in grey.

- Molecule 1: Actin

Chain A:

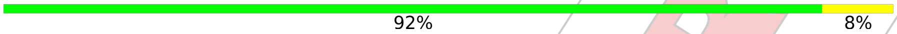

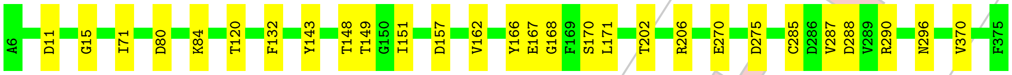

- Molecule 1: Actin

Chain B:

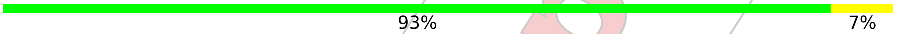

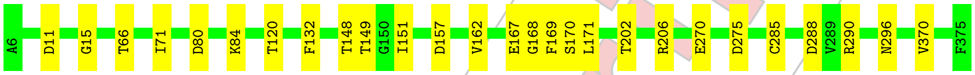

- Molecule 1: Actin

Chain C:

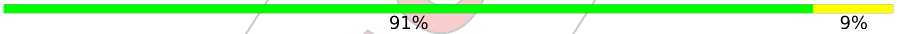

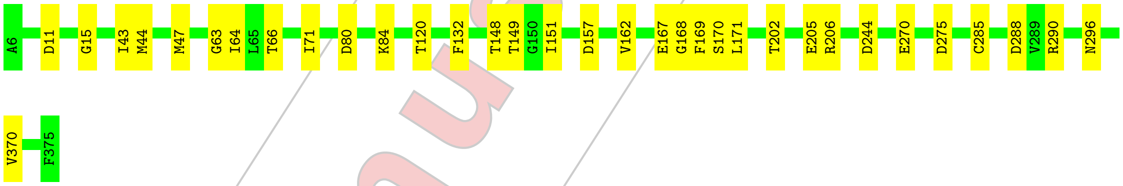

- Molecule 1: Actin

Chain D:

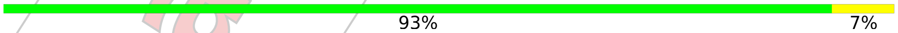

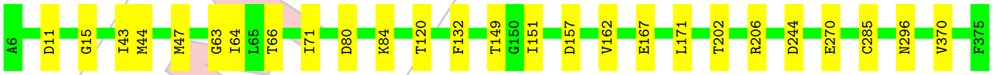

- Molecule 1: Actin

Chain E:

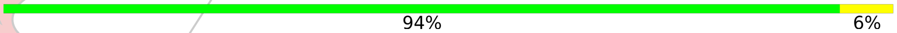

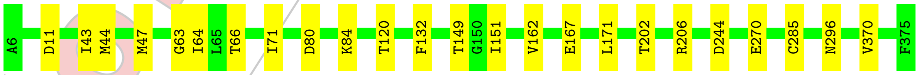

For Manuscript Review

4 Experimental information ⓘ

Property	Value	Source
EM reconstruction method	HELICAL	Depositor
Imposed symmetry	HELICAL, twist=-167.25°, rise=27.49 Å, axial sym=C1	Depositor
Number of segments used	195534	Depositor
Resolution determination method	FSC 0.143 CUT-OFF	Depositor
CTF correction method	PHASE FLIPPING AND AMPLITUDE CORRECTION	Depositor
Microscope	FEI TITAN KRIOS	Depositor
Voltage (kV)	300	Depositor
Electron dose ($e^-/\text{Å}^2$)	60	Depositor
Minimum defocus (nm)	800	Depositor
Maximum defocus (nm)	2800	Depositor
Magnification	Not provided	
Image detector	FEI FALCON II (4k x 4k)	Depositor

For Manuscript Review

5 Model quality (i)

5.1 Standard geometry (i)

Bond lengths and bond angles in the following residue types are not validated in this section: MG, ADP

The Z score for a bond length (or angle) is the number of standard deviations the observed value is removed from the expected value. A bond length (or angle) with $|Z| > 5$ is considered an outlier worth inspection. RMSZ is the root-mean-square of all Z scores of the bond lengths (or angles).

Mol	Chain	Bond lengths		Bond angles	
		RMSZ	#|Z| >5	RMSZ	#|Z| >5
1	A	0.32	0/2956	0.55	0/4000
1	B	0.32	0/2956	0.55	0/4000
1	C	0.32	0/2956	0.55	0/4000
1	D	0.32	0/2956	0.55	0/4000
1	E	0.32	0/2956	0.54	0/4000
All	All	0.32	0/14780	0.55	0/20000

There are no bond length outliers.

There are no bond angle outliers.

There are no chirality outliers.

There are no planarity outliers.

5.2 Too-close contacts (i)

In the following table, the Non-H and H(model) columns list the number of non-hydrogen atoms and hydrogen atoms in the chain respectively. The H(added) column lists the number of hydrogen atoms added and optimized by MolProbity. The Clashes column lists the number of clashes within the asymmetric unit, whereas Symm-Clashes lists symmetry-related clashes.

Mol	Chain	Non-H	H(model)	H(added)	Clashes	Symm-Clashes
1	A	2892	0	2860	30	0
1	B	2892	0	2860	27	0
1	C	2892	0	2860	42	0
1	D	2892	0	2860	27	0
1	E	2892	0	2860	25	0
2	A	27	0	12	0	0
2	B	27	0	12	0	0
2	C	27	0	12	0	0

Continued on next page...

For Manuscript Review

Continued from previous page...

Mol	Chain	Non-H	H(model)	H(added)	Clashes	Symm-Clashes
2	D	27	0	12	0	0
2	E	27	0	12	0	0
3	A	1	0	0	0	0
3	B	1	0	0	0	0
3	C	1	0	0	0	0
3	D	1	0	0	0	0
3	E	1	0	0	0	0
All	All	14600	0	14360	111	0

The all-atom clashscore is defined as the number of clashes found per 1000 atoms (including hydrogen atoms). The all-atom clashscore for this structure is 4.

All (111) close contacts within the same asymmetric unit are listed below, sorted by their clash magnitude.

Atom-1	Atom-2	Interatomic distance (Å)	Clash overlap (Å)
1:A:288:ASP:OD2	1:C:63:GLY:N	1.89	1.05
1:B:288:ASP:OD2	1:D:63:GLY:N	2.01	0.93
1:C:288:ASP:OD2	1:E:63:GLY:N	2.01	0.93
1:B:120:THR:HG21	1:B:370:VAL:HG21	1.65	0.79
1:D:120:THR:HG21	1:D:370:VAL:HG21	1.65	0.78
1:A:120:THR:HG21	1:A:370:VAL:HG21	1.65	0.78
1:C:120:THR:HG21	1:C:370:VAL:HG21	1.65	0.77
1:E:120:THR:HG21	1:E:370:VAL:HG21	1.65	0.77
1:C:290:ARG:NH2	1:E:244:ASP:OD2	2.18	0.76
1:B:290:ARG:NH2	1:D:244:ASP:OD2	2.19	0.75
1:E:80:ASP:OD2	1:E:84:LYS:NZ	2.26	0.68
1:A:288:ASP:OD2	1:C:63:GLY:CA	2.42	0.68
1:C:80:ASP:OD2	1:C:84:LYS:NZ	2.26	0.67
1:A:290:ARG:NH2	1:C:244:ASP:OD2	2.28	0.66
1:A:80:ASP:OD2	1:A:84:LYS:NZ	2.26	0.66
1:A:120:THR:HG22	1:A:132:PHE:CE2	2.31	0.65
1:C:120:THR:HG22	1:C:132:PHE:CE2	2.31	0.65
1:D:80:ASP:OD2	1:D:84:LYS:NZ	2.26	0.65
1:E:120:THR:HG22	1:E:132:PHE:CE2	2.32	0.65
1:D:120:THR:HG22	1:D:132:PHE:CE2	2.31	0.65
1:B:120:THR:HG22	1:B:132:PHE:CE2	2.32	0.65
1:B:80:ASP:OD2	1:B:84:LYS:NZ	2.26	0.64
1:B:170:SER:HB3	1:D:43:ILE:HD13	1.81	0.62
1:A:148:THR:HG21	1:C:47:MET:SD	2.40	0.62
1:A:170:SER:HB3	1:C:43:ILE:HD13	1.81	0.62
1:C:170:SER:HB3	1:E:43:ILE:HD13	1.83	0.60

Continued on next page...

For Manuscript Review

Continued from previous page...

Atom-1	Atom-2	Interatomic distance (Å)	Clash overlap (Å)
1:A:288:ASP:HB3	1:C:63:GLY:HA3	1.85	0.59
1:B:148:THR:HG21	1:D:47:MET:SD	2.44	0.58
1:A:168:GLY:HA2	1:C:44:MET:HG3	1.86	0.58
1:D:270:GLU:O	1:D:270:GLU:HG3	2.04	0.57
1:E:270:GLU:O	1:E:270:GLU:HG3	2.04	0.57
1:C:270:GLU:O	1:C:270:GLU:HG3	2.04	0.57
1:C:148:THR:HG21	1:E:47:MET:SD	2.44	0.57
1:A:270:GLU:O	1:A:270:GLU:HG3	2.04	0.57
1:C:288:ASP:OD2	1:E:63:GLY:CA	2.53	0.57
1:B:270:GLU:O	1:B:270:GLU:HG3	2.04	0.57
1:B:288:ASP:OD2	1:D:63:GLY:CA	2.53	0.56
1:C:168:GLY:HA2	1:E:44:MET:HG3	1.89	0.55
1:D:149:THR:HG22	1:D:167:GLU:H	1.72	0.55
1:E:149:THR:HG22	1:E:167:GLU:H	1.72	0.54
1:A:149:THR:HG22	1:A:167:GLU:H	1.72	0.54
1:B:168:GLY:HA2	1:D:44:MET:HG3	1.88	0.54
1:B:149:THR:HG22	1:B:167:GLU:H	1.72	0.53
1:B:288:ASP:HB3	1:D:63:GLY:HA3	1.90	0.53
1:C:149:THR:HG22	1:C:167:GLU:H	1.72	0.53
1:C:288:ASP:HB3	1:E:63:GLY:HA3	1.91	0.53
1:A:149:THR:O	1:A:296:ASN:ND2	2.44	0.51
1:E:202:THR:O	1:E:206:ARG:N	2.40	0.51
1:C:202:THR:O	1:C:206:ARG:N	2.40	0.50
1:E:149:THR:O	1:E:296:ASN:ND2	2.44	0.50
1:A:288:ASP:HB3	1:C:63:GLY:CA	2.42	0.49
1:D:149:THR:O	1:D:296:ASN:ND2	2.44	0.49
1:A:202:THR:O	1:A:206:ARG:N	2.40	0.49
1:D:171:LEU:HD23	1:D:285:CYS:HB2	1.95	0.49
1:E:171:LEU:HD23	1:E:285:CYS:HB2	1.95	0.49
1:B:171:LEU:HD23	1:B:285:CYS:HB2	1.95	0.49
1:C:171:LEU:HD23	1:C:285:CYS:HB2	1.95	0.48
1:A:171:LEU:HD23	1:A:285:CYS:HB2	1.95	0.48
1:C:149:THR:O	1:C:296:ASN:ND2	2.44	0.48
1:B:202:THR:O	1:B:206:ARG:N	2.40	0.47
1:D:120:THR:HG22	1:D:132:PHE:HE2	1.79	0.46
1:B:149:THR:O	1:B:296:ASN:ND2	2.44	0.46
1:C:270:GLU:OE1	1:D:66:THR:HG22	2.16	0.46
1:C:169:PHE:CE2	1:E:64:ILE:CD1	2.99	0.45
1:A:270:GLU:OE1	1:B:66:THR:HG22	2.17	0.45
1:B:120:THR:HG22	1:B:132:PHE:HE2	1.79	0.44
1:B:169:PHE:CE2	1:D:64:ILE:CD1	3.00	0.44

Continued on next page...

For Manuscript Review

Continued from previous page...

Atom-1	Atom-2	Interatomic distance (Å)	Clash overlap (Å)
1:B:270:GLU:OE1	1:C:66:THR:HG22	2.16	0.44
1:A:170:SER:HB3	1:C:43:ILE:HG21	1.98	0.44
1:D:202:THR:O	1:D:206:ARG:N	2.40	0.44
1:A:120:THR:HG22	1:A:132:PHE:HE2	1.79	0.43
1:A:166:TYR:HD2	1:C:64:ILE:HG21	1.84	0.43
1:A:288:ASP:CB	1:C:63:GLY:CA	2.96	0.43
1:C:120:THR:HG22	1:C:132:PHE:HE2	1.79	0.43
1:D:120:THR:HG21	1:D:370:VAL:CG2	2.43	0.43
1:A:120:THR:HG21	1:A:370:VAL:CG2	2.43	0.43
1:A:148:THR:CG2	1:C:47:MET:SD	3.05	0.42
1:D:270:GLU:OE1	1:E:66:THR:HG22	2.19	0.42
1:B:170:SER:HB3	1:D:43:ILE:HG21	2.01	0.42
1:D:151:ILE:HD11	1:D:162:VAL:CG1	2.50	0.42
1:E:71:ILE:O	1:E:71:ILE:HG23	2.20	0.42
1:B:151:ILE:HD11	1:B:162:VAL:CG1	2.50	0.42
1:B:71:ILE:HG23	1:B:71:ILE:O	2.20	0.42
1:B:288:ASP:HB3	1:D:63:GLY:CA	2.49	0.42
1:C:120:THR:HG21	1:C:370:VAL:CG2	2.43	0.42
1:D:71:ILE:HG23	1:D:71:ILE:O	2.20	0.42
1:E:120:THR:HG22	1:E:132:PHE:HE2	1.79	0.42
1:C:11:ASP:OD1	1:C:11:ASP:O	2.38	0.41
1:E:151:ILE:HD11	1:E:162:VAL:CG1	2.50	0.41
1:A:71:ILE:O	1:A:71:ILE:HG23	2.20	0.41
1:A:11:ASP:O	1:A:11:ASP:OD1	2.38	0.41
1:C:151:ILE:HD11	1:C:162:VAL:CG1	2.50	0.41
1:D:11:ASP:OD1	1:D:11:ASP:O	2.38	0.41
1:C:71:ILE:HG23	1:C:71:ILE:O	2.20	0.41
1:C:148:THR:CG2	1:E:47:MET:SD	3.09	0.41
1:E:120:THR:HG21	1:E:370:VAL:CG2	2.43	0.41
1:B:11:ASP:O	1:B:11:ASP:OD1	2.38	0.41
1:B:148:THR:CG2	1:D:47:MET:SD	3.09	0.41
1:A:151:ILE:HD11	1:A:162:VAL:CG1	2.50	0.41
1:B:15:GLY:HA3	1:B:157:ASP:OD2	2.21	0.41
1:B:275:ASP:OD1	1:B:275:ASP:N	2.54	0.41
1:A:275:ASP:N	1:A:275:ASP:OD1	2.54	0.41
1:C:288:ASP:HB3	1:E:63:GLY:CA	2.50	0.41
1:A:287:VAL:HG21	1:C:205:GLU:OE2	2.21	0.40
1:E:11:ASP:O	1:E:11:ASP:OD1	2.38	0.40
1:A:143:TYR:CE2	1:C:44:MET:HE3	2.56	0.40
1:D:15:GLY:HA3	1:D:157:ASP:OD2	2.21	0.40
1:A:15:GLY:HA3	1:A:157:ASP:OD2	2.21	0.40

Continued on next page...

For Manuscript Review

Continued from previous page...

Atom-1	Atom-2	Interatomic distance (Å)	Clash overlap (Å)
1:C:15:GLY:HA3	1:C:157:ASP:OD2	2.21	0.40
1:C:170:SER:HB3	1:E:43:ILE:HG21	2.03	0.40
1:C:275:ASP:N	1:C:275:ASP:OD1	2.54	0.40

There are no symmetry-related clashes.

5.3 Torsion angles (i)

5.3.1 Protein backbone (i)

In the following table, the Percentiles column shows the percent Ramachandran outliers of the chain as a percentile score with respect to all PDB entries followed by that with respect to all EM entries.

The Analysed column shows the number of residues for which the backbone conformation was analysed, and the total number of residues.

Mol	Chain	Analysed	Favoured	Allowed	Outliers	Percentiles	
1	A	368/370 (100%)	360 (98%)	8 (2%)	0	100	100
1	B	368/370 (100%)	360 (98%)	8 (2%)	0	100	100
1	C	368/370 (100%)	360 (98%)	8 (2%)	0	100	100
1	D	368/370 (100%)	360 (98%)	8 (2%)	0	100	100
1	E	368/370 (100%)	360 (98%)	8 (2%)	0	100	100
All	All	1840/1850 (100%)	1800 (98%)	40 (2%)	0	100	100

There are no Ramachandran outliers to report.

5.3.2 Protein sidechains (i)

In the following table, the Percentiles column shows the percent sidechain outliers of the chain as a percentile score with respect to all PDB entries followed by that with respect to all EM entries.

The Analysed column shows the number of residues for which the sidechain conformation was analysed, and the total number of residues.

Mol	Chain	Analysed	Rotameric	Outliers	Percentiles	
1	A	316/316 (100%)	316 (100%)	0	100	100

Continued on next page...

For Manuscript Review

Continued from previous page...

Mol	Chain	Analysed	Rotameric	Outliers	Percentiles	
1	B	316/316 (100%)	316 (100%)	0	100	100
1	C	316/316 (100%)	316 (100%)	0	100	100
1	D	316/316 (100%)	316 (100%)	0	100	100
1	E	316/316 (100%)	316 (100%)	0	100	100
All	All	1580/1580 (100%)	1580 (100%)	0	100	100

There are no protein residues with a non-rotameric sidechain to report.

Sometimes sidechains can be flipped to improve hydrogen bonding and reduce clashes. There are no such sidechains identified.

5.3.3 RNA (i)

There are no RNA molecules in this entry.

5.4 Non-standard residues in protein, DNA, RNA chains (i)

There are no non-standard protein/DNA/RNA residues in this entry.

5.5 Carbohydrates (i)

There are no monosaccharides in this entry.

5.6 Ligand geometry (i)

Of 10 ligands modelled in this entry, 5 are monoatomic - leaving 5 for Mogul analysis.

In the following table, the Counts columns list the number of bonds (or angles) for which Mogul statistics could be retrieved, the number of bonds (or angles) that are observed in the model and the number of bonds (or angles) that are defined in the Chemical Component Dictionary. The Link column lists molecule types, if any, to which the group is linked. The Z score for a bond length (or angle) is the number of standard deviations the observed value is removed from the expected value. A bond length (or angle) with $|Z| > 2$ is considered an outlier worth inspection. RMSZ is the root-mean-square of all Z scores of the bond lengths (or angles).

Mol	Type	Chain	Res	Link	Bond lengths			Bond angles		
					Counts	RMSZ	$\#\|Z\| > 2$	Counts	RMSZ	$\#\|Z\| > 2$
2	ADP	A	401	3	24,29,29	0.97	1 (4%)	29,45,45	1.41	3 (10%)
2	ADP	E	401	3	24,29,29	0.96	1 (4%)	29,45,45	1.41	3 (10%)

For Manuscript Review

Mol	Type	Chain	Res	Link	Bond lengths			Bond angles		
					Counts	RMSZ	#|Z| > 2	Counts	RMSZ	#|Z| > 2
2	ADP	B	401	3	24,29,29	0.97	1 (4%)	29,45,45	1.42	3 (10%)
2	ADP	D	401	3	24,29,29	0.97	1 (4%)	29,45,45	1.41	3 (10%)
2	ADP	C	401	3	24,29,29	0.96	1 (4%)	29,45,45	1.41	3 (10%)

In the following table, the Chirals column lists the number of chiral outliers, the number of chiral centers analysed, the number of these observed in the model and the number defined in the Chemical Component Dictionary. Similar counts are reported in the Torsion and Rings columns. '-' means no outliers of that kind were identified.

Mol	Type	Chain	Res	Link	Chirals	Torsions	Rings
2	ADP	A	401	3	-	2/12/32/32	0/3/3/3
2	ADP	E	401	3	-	2/12/32/32	0/3/3/3
2	ADP	B	401	3	-	2/12/32/32	0/3/3/3
2	ADP	D	401	3	-	2/12/32/32	0/3/3/3
2	ADP	C	401	3	-	2/12/32/32	0/3/3/3

All (5) bond length outliers are listed below:

Mol	Chain	Res	Type	Atoms	Z	Observed(Å)	Ideal(Å)
2	D	401	ADP	C5-C4	2.36	1.47	1.40
2	B	401	ADP	C5-C4	2.34	1.47	1.40
2	A	401	ADP	C5-C4	2.33	1.47	1.40
2	E	401	ADP	C5-C4	2.32	1.47	1.40
2	C	401	ADP	C5-C4	2.27	1.46	1.40

All (15) bond angle outliers are listed below:

Mol	Chain	Res	Type	Atoms	Z	Observed(^{o})	Ideal(^{o})
2	B	401	ADP	PA-O3A-PB	-4.27	118.18	132.83
2	A	401	ADP	PA-O3A-PB	-4.25	118.23	132.83
2	E	401	ADP	PA-O3A-PB	-4.25	118.25	132.83
2	C	401	ADP	PA-O3A-PB	-4.24	118.29	132.83
2	D	401	ADP	PA-O3A-PB	-4.23	118.31	132.83
2	D	401	ADP	N3-C2-N1	-3.41	123.35	128.68
2	A	401	ADP	N3-C2-N1	-3.41	123.36	128.68
2	B	401	ADP	N3-C2-N1	-3.41	123.36	128.68
2	C	401	ADP	N3-C2-N1	-3.39	123.38	128.68
2	E	401	ADP	N3-C2-N1	-3.38	123.39	128.68
2	B	401	ADP	C4-C5-N7	-2.48	106.81	109.40
2	C	401	ADP	C4-C5-N7	-2.42	106.88	109.40

Continued on next page...

For Manuscript Review

Continued from previous page...

Mol	Chain	Res	Type	Atoms	Z	Observed(^{o})	Ideal(^{o})
2	E	401	ADP	C4-C5-N7	-2.42	106.88	109.40
2	A	401	ADP	C4-C5-N7	-2.40	106.89	109.40
2	D	401	ADP	C4-C5-N7	-2.37	106.92	109.40

There are no chirality outliers.

All (10) torsion outliers are listed below:

Mol	Chain	Res	Type	Atoms
2	A	401	ADP	C5'-O5'-PA-O2A
2	A	401	ADP	C5'-O5'-PA-O3A
2	B	401	ADP	C5'-O5'-PA-O2A
2	B	401	ADP	C5'-O5'-PA-O3A
2	C	401	ADP	C5'-O5'-PA-O2A
2	C	401	ADP	C5'-O5'-PA-O3A
2	D	401	ADP	C5'-O5'-PA-O2A
2	D	401	ADP	C5'-O5'-PA-O3A
2	E	401	ADP	C5'-O5'-PA-O2A
2	E	401	ADP	C5'-O5'-PA-O3A

There are no ring outliers.

No monomer is involved in short contacts.

The following is a two-dimensional graphical depiction of Mogul quality analysis of bond lengths, bond angles, torsion angles, and ring geometry for all instances of the Ligand of Interest. In addition, ligands with molecular weight > 250 and outliers as shown on the validation Tables will also be included. For torsion angles, if less then 5% of the Mogul distribution of torsion angles is within 10 degrees of the torsion angle in question, then that torsion angle is considered an outlier. Any bond that is central to one or more torsion angles identified as an outlier by Mogul will be highlighted in the graph. For rings, the root-mean-square deviation (RMSD) between the ring in question and similar rings identified by Mogul is calculated over all ring torsion angles. If the average RMSD is greater than 60 degrees and the minimal RMSD between the ring in question and any Mogul-identified rings is also greater than 60 degrees, then that ring is considered an outlier. The outliers are highlighted in purple. The color gray indicates Mogul did not find sufficient equivalents in the CSD to analyse the geometry.

For Manuscript Review

Ligand ADP A 401

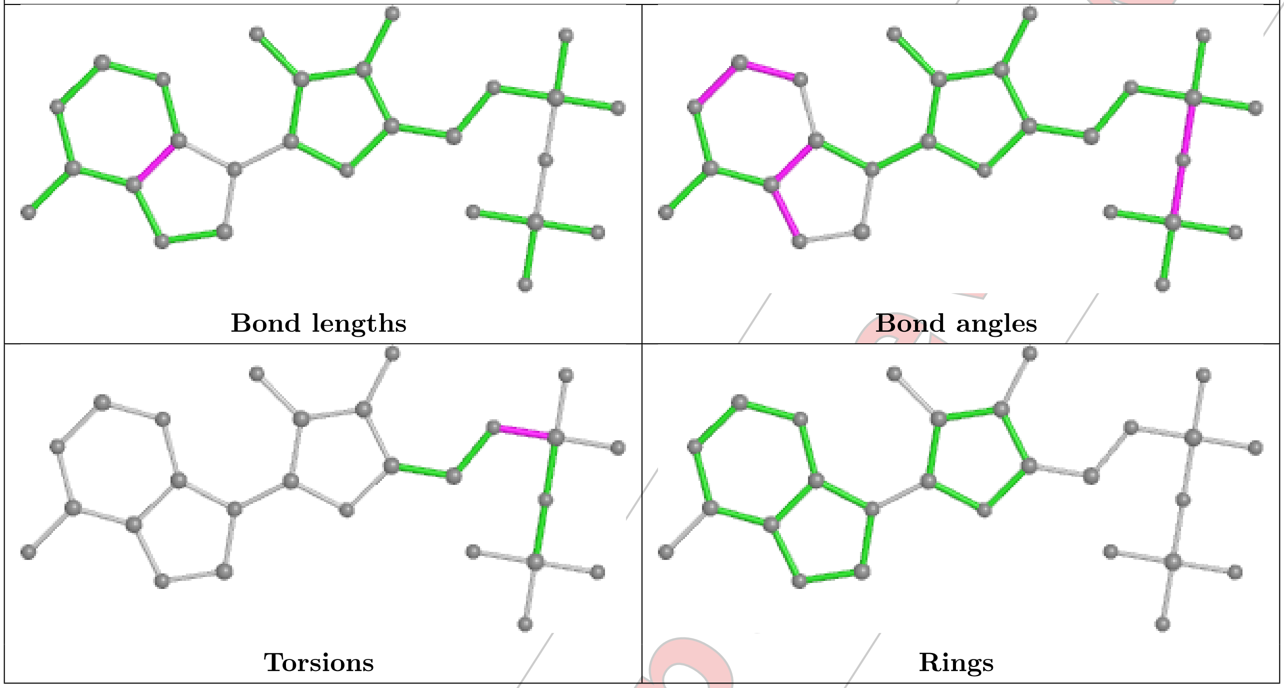

Ligand ADP E 401

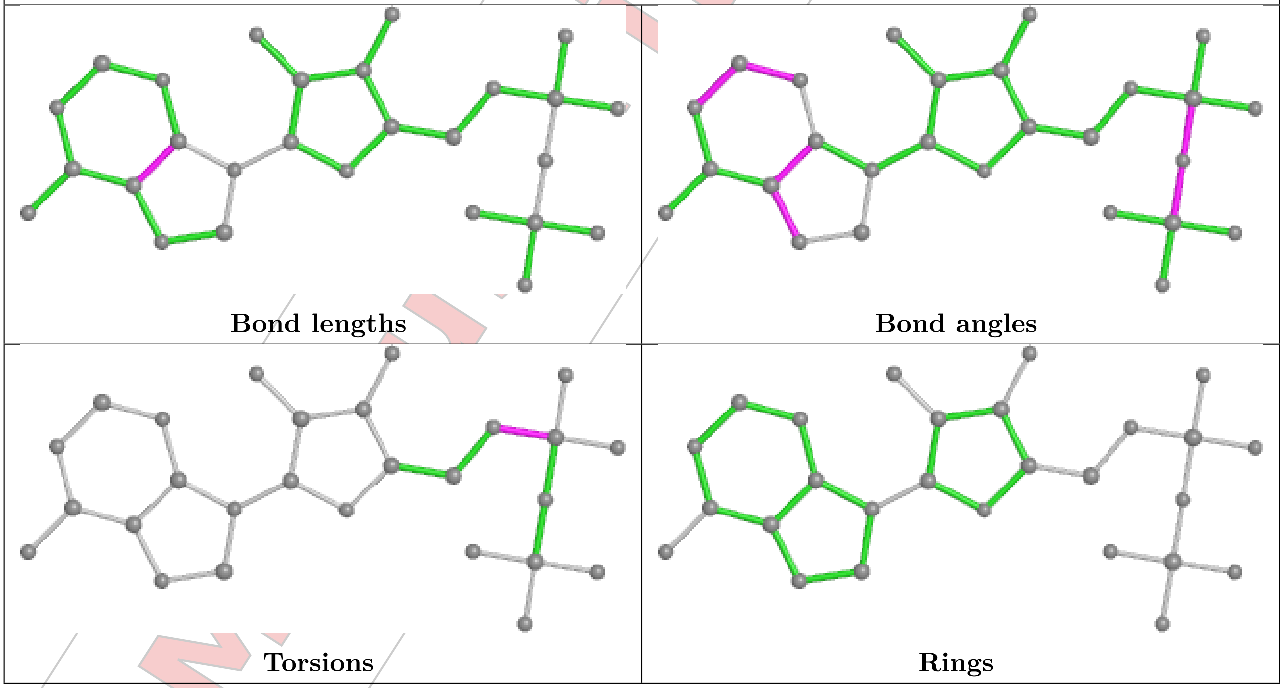

Ligand ADP B 401

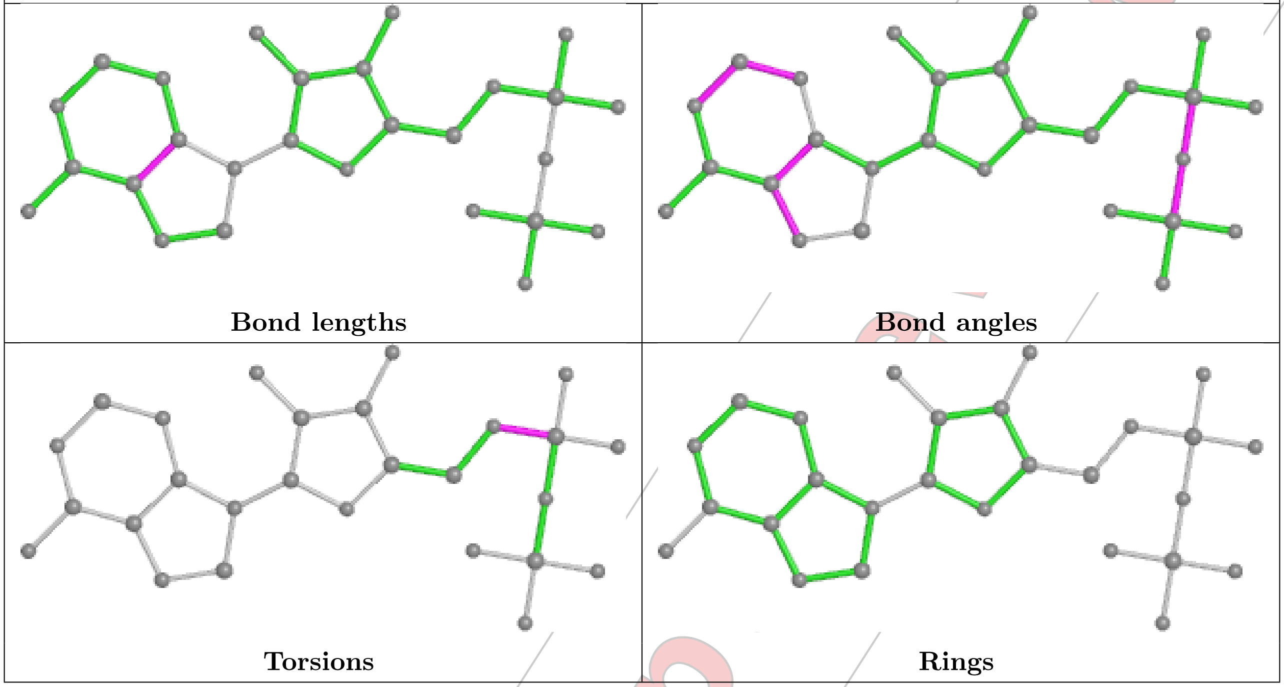

Ligand ADP D 401

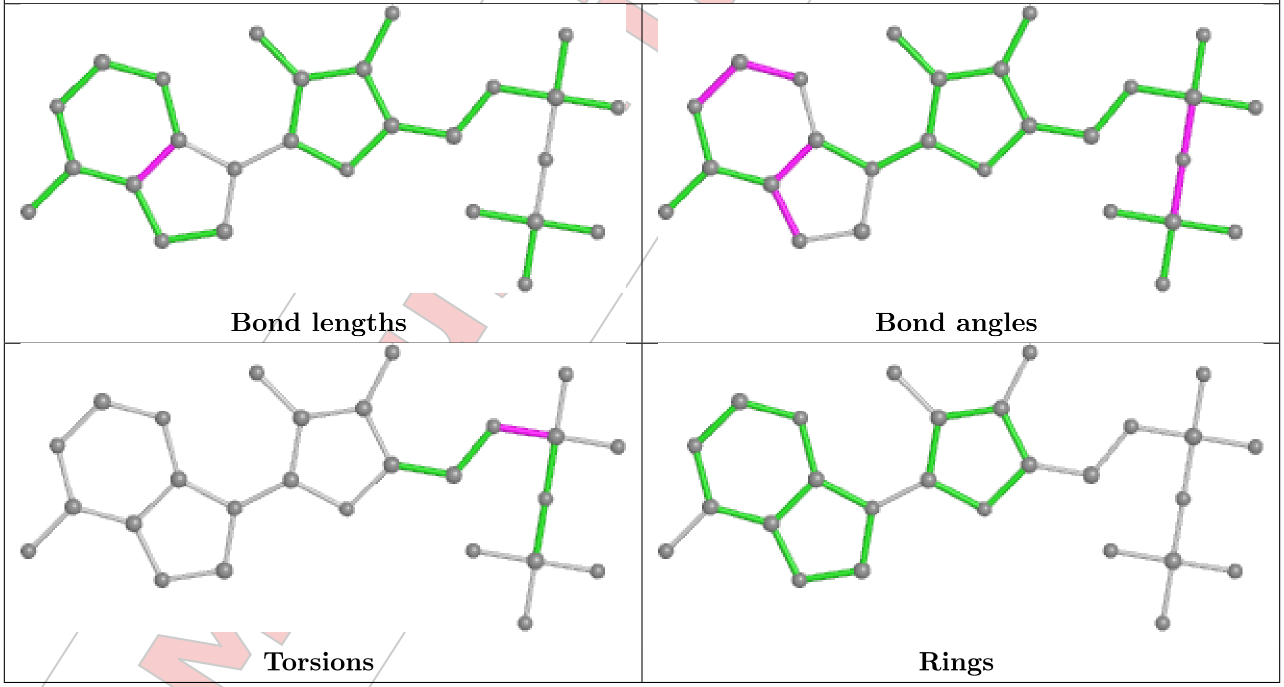

Ligand ADP C 401

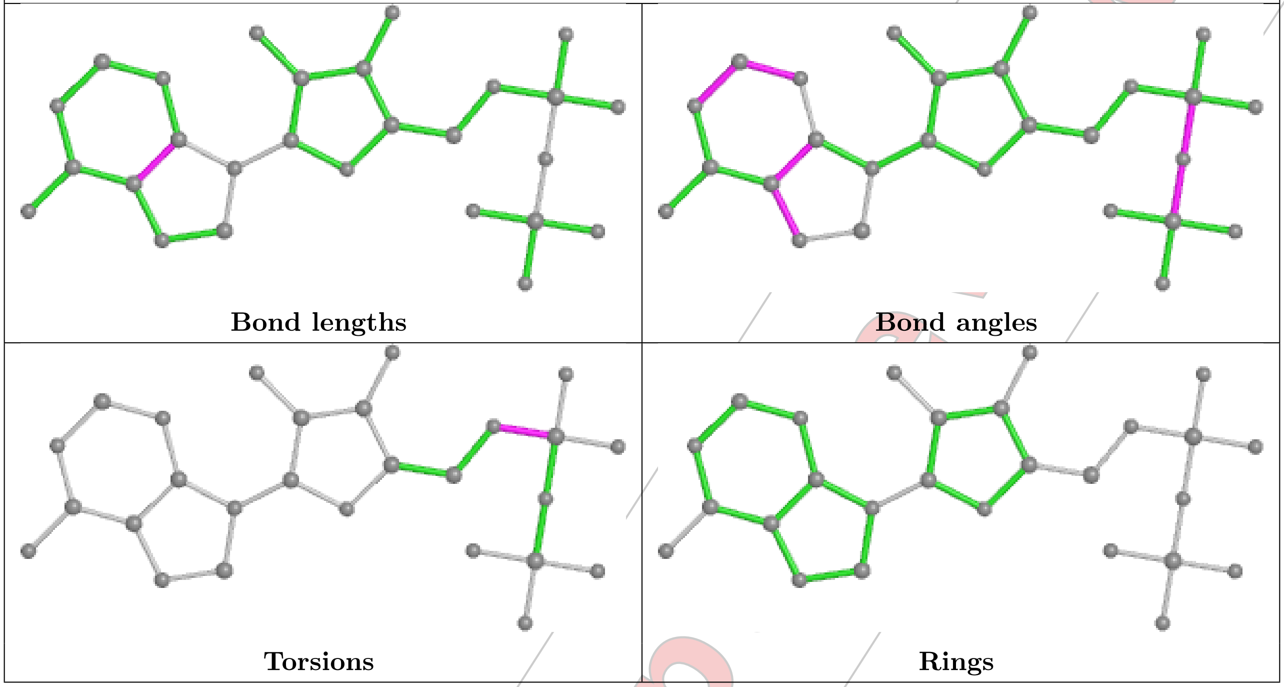

5.7 Other polymers (i)

There are no such residues in this entry.

5.8 Polymer linkage issues (i)

There are no chain breaks in this entry.

For Manuscript Review
